# The transcriptome of *Icerya aegyptiaca* (Hemiptera: Monophlebidae) and comparison with neococcoids reveal genetic clues of evolution in the scale insects

**DOI:** 10.1186/s12864-023-09327-z

**Published:** 2023-05-03

**Authors:** Xue-Fei Tang, Yu-Hao Huang, Yi-Fei Sun, Pei-Fang Zhang, Li-Zhi Huo, Hao-Sen Li, Hong Pang

**Affiliations:** 1 State Key Laboratory of Biocontrol, School of Ecology, Sun Yat-Sen University, Shenzhen, China; 2 Guangzhou Institute of Forestry and Landscape Architecture, Guangzhou, China

**Keywords:** Transcriptome, *Icerya aegyptiaca*, Scale insects, Neococcoids, Evolution

## Abstract

**Background:**

Scale insects are worldwide sap-sucking parasites, which can be distinguished into neococcoids and non-neococcoids. Neococcoids are monophyletic with a peculiar reproductive system, paternal genome elimination (PGE). Different with neococcoids, Iceryini, a tribe in non-neococcoids including several damaging pests, has abdominal spiracles, compound eyes in males, relatively abundant wax, unique hermaphrodite system, and specific symbionts. However, the current studies on the gene resources and genomic mechanism of scale insects are mainly limited in the neococcoids, and lacked of comparison in an evolution frame.

**Result:**

We sequenced and de novo assembled a transcriptome of *Icerya aegyptiaca* (Douglas), a worldwide pest of Iceryini, and used it as representative of non-neococcoids to compare with the genomes or transcriptomes of other six species from different families of neococcoids. We found that the genes under positive selection or negative selection intensification (simplified as “selected genes” below) in *I. aegyptiaca* included those related to neurogenesis and development, especially eye development. Some genes related to fatty acid biosynthesis were unique in its transcriptome with relatively high expression and not detected in neococcoids. These results may indicate a potential link to the unique structures and abundant wax of *I. aegyptiaca* compared with neococcoids. Meanwhile, genes related to DNA repair, mitosis, spindle, cytokinesis and oogenesis, were included in the selected genes in *I. aegyptiaca*, which is possibly associated with cell division and germ cell formation of the hermaphrodite system. Chromatin-related process were enriched from selected genes in neococcoids, along with some mitosis-related genes also detected, which may be related to their unique PGE system. Moreover, in neococcoid species, male-biased genes tend to undergo negative selection relaxation under the PGE system. We also found that the candidate horizontally transferred genes (HTGs) in the scale insects mainly derived from bacteria and fungi. *bioD* and *bioB*, the two biotin-synthesizing HTGs were exclusively found in the scale insects and neococcoids, respectively, which possibly show potential demand changes in the symbiotic relationships.

**Conclusion:**

Our study reports the first *I. aegyptiaca* transcriptome and provides preliminary insights for the genetic change of structures, reproductive systems and symbiont relationships at an evolutionary aspect. This will provide a basis for further research and control of scale insects.

**Supplementary Information:**

The online version contains supplementary material available at 10.1186/s12864-023-09327-z.

## Introduction

Scale insects (Hemiptera: Coccoidea) are sap-sucking plant parasites distributed worldwide, including at least 8,194 species among 50 families [1]. They are severe pests of fruit and nut trees, forest vegetation, woody ornamentals, greenhouse and indoor plants [2]. They caused catastrophic damage to the plants primarily through tapping into the phloem directly to ingest the plant sap or indirectly excreting honeydew, which will lead to mould contamination [3]. However, scale insects can also play positive roles in ecosystems, including human beings. For example, Dactylopiidae, also known as the cochineal insects, is sources of dyestuff [4]. And the lac scales (Kerriidae) have been used by humans since early times for their commercial products, such as lacdye, lac wax and lac resin [5].

Scale insects are generally divided into two informal groups, archaeococcoids and neococcoids, sometimes treated as superfamilies [6, 7]. In current phylogenetic studies, the monophyly of neococcoids, mainly including Pseudococcidae (mealybugs), Aclerdidae, Coccidae, Diaspididae, Eriococcidae, Kermesidae, Kerriidae and Dactylopiidae, has been confirmed [8, 9]. For example, the pink hibiscus mealybug *Maconellicoccus hirsutus* (Hemiptera: Pseudococcidae), which feeds on several host plants and causes severe economic lost in tropical and subtropical regions [10], and the Chinese white wax scale insect *Ericerus pela* (Hemiptera: Coccidae), famous and bred in China due to its wax able to be used in printing, candle production, and traditional medicine [11], are representative members of neococcoids. The species in neococcoids always lack abdominal spiracles and compound eyes in males [12], and process a special reproductive system, paternal genome elimination (PGE) [13], while most species of non-neococcoids have abdominal spiracles and compound eyes in males, and an XX-XO reproductive system, which are plesiomorphies in the Hemiptera [6, 14].

Actually, the scale insects possess diverse range of sex determination mechanisms and chromosome behaviors [14], probably related to their inverse meiosis and holokinetic chromosomes [15, 16]. The reproductive systems found in the scale insects now include 10 types, including hermaphroditism and PGE [6, 13]. Hermaphroditism is extreme scarce in insects relative to their huge populations [5]. The only known cases of hermaphroditism in insects are found in Iceryini, including *Icerya purchasi* Maskell and *I. aegyptiaca* (Douglas) [17, 18]. Furthermore, the study of population genetic analysis supported the possibility of *I. purchasi* to be hermaphrodite or even androdioecy, which means the coexistence of males and hermaphrodites with a modified gonad structure that produces both sperm and eggs [17]. In *I. aegyptiaca*, it was also found that the reproductive system was haplodiploidy and hermaphrodite, with a gonad structure called “ovotestis” in female [18]. PGE, which is also one of the most peculiar reproductive systems in the scale insects, exists in most neococcoid species, with a single origin [6]. Both their sexes develop from fertilized eggs in PGE system, but the male offspring will gradually loss the half of the paternal genome during early development by heterochromatinization [19]. However, definitive explanations for such diverse reproductive systems are still scarce.

Further, scale insects have different symbiotic bacteria (e.g. Flavobacteriales in Iceryini [20–22] and *Tremblaya***/***Moranella* in mealybugs (Pseudococcidae) [23–25]). They feed exclusively on phloem of their host plant, which is rich in sugars but poor in amino acids and vitamins, with the symbiotic bacteria in bacteriocytes of scale insects as important providers of these essential nutrients [1, 26]. On the other hand, scale insects obtain genes from these symbiotic bacteria or other bacteria and express these horizontally transferred genes (HTGs) in their bacteriocytes, to complement the missing parts of the nutrient and structural synthesis pathways in these symbiotic bacteria with reduced genomes [23, 27–29], which indicates a highly close symbiotic relationship. Moreover, some maternally inherited symbiotic bacteria (e.g. *Wolbachia*) may contribute to hermaphroditism of Iceryini [30]. Therefore, it is important to realize the symbiotic relationships and their evolution in the scale insects. However, except the mealybugs, the symbiotic relationships of the scale insects, especially the HTGs, are largely unknown.

**Fig. 1:**
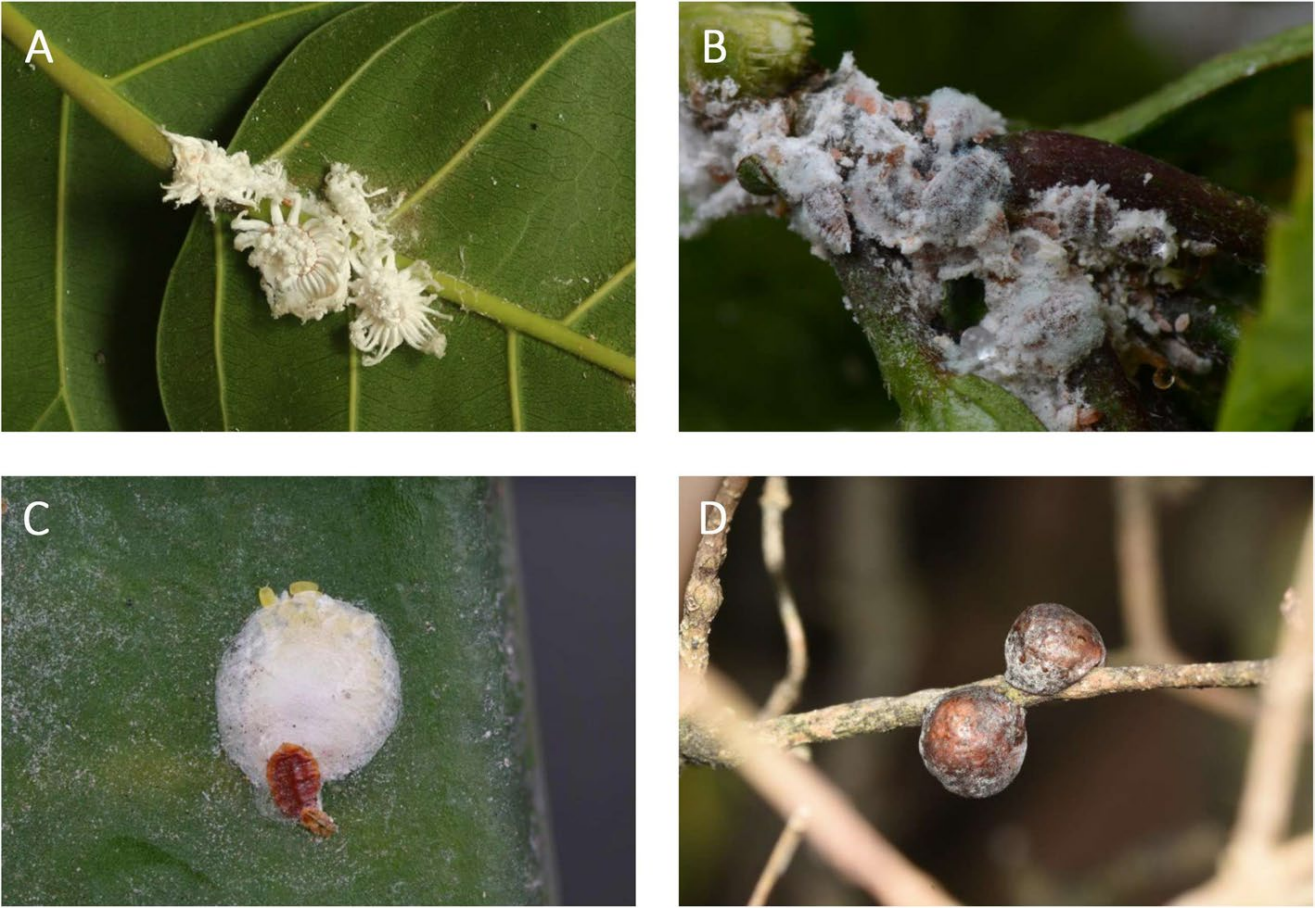
The female of the scale insects. **A** *Icerya aegyptiaca* (Monophlebidae). Neococcoids: **B** *Maconellicoccus hirsutus* (Pseudococcidae). **C** *Pseudaulacaspis cockerelli* (Diaspididae). **D** *Ericerus pela* (Coccidae)

The Egyptian cottony cushion scale, *I. aegyptiaca* (Douglas), is a globally distributed invasive pest belonging to tribe Iceryini (Coccoidea: Monophlebidae) and archaeococcids. It attacks more than 200 species of plants, excretes honeydew, causes the growth of sooty mold on plants and attracts the invasive ant species, which results in big losses in agriculture annually [31]. Different with neococcoids, *I. aegyptiaca* has several unique characters of adult females, such as rare genetic system (hermaphrodite), specific symbiotic bacteria and waxy filaments extended from their bodies (Fig. 1). They protect themselves with the formation of a hydrophobicity wax shell, which is impenetrable even by chemical insecticides [32]. But the mechanisms of forming these characters are far from known. The genetic resource of *I. aegyptiaca* can be helpful to explore these mechanisms and serves as a representative of non-neococcoid species to explore the evolution of whole scale lineages.

In this study, we sequenced and assembled the transcriptome of *I. aegyptiaca*. By comparing with genome and transcriptome of six species from different neococcoid families, we identified the highly expressed genes unique in *I. aegyptiaca*, detected the selection pressure, and examined the horizontally transferred genes, which provides genetic clues for the formation mechanism of structures, reproductive systems and symbiotic relationships.

## Results

### General features of transcriptome of *I. aegyptiaca*

For each of all the four samples, 7.01–7.68 Gb clean data was generated by Illumina platform. The transcriptome of *I. aegyptiaca* was assembled by Trinity [33] into 235,635 contigs with 33.88% transcriptome-wide GC content and the N50 of 2,076 bp. Protein set generated by EvidentialGene [34] includes 72,003 proteins, 13,805 of which were annotated in InterPro [35, 36], 15,793 in Eggnog [37, 38] and 9,828 in Gene Ontology (GO) [39] database. Benchmarking Universal Single-Copy Orthologs (BUSCO) pipeline [40] detects 92.7% complete genes (90.0% single-copy and 2.7% duplicated genes), 1.0% fragment, 6.3% missing genes from the protein set, indicating a relatively high integrity.

### General features of orthologous groups

**Fig. 2:**
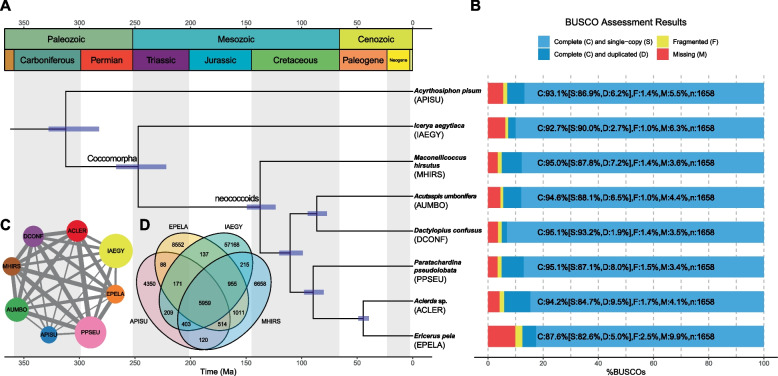
Phylogeny and status of orthologous groups (OGs) based on the protein sets of seven scale insects and one outgroup aphid species. **A** The species tree and estimated divergence time. The phylogeny was obtained by IQ-TREE inference with all the fully supported inter nodes (ultrafast bootstrap (UFBoot) support values = 100) and consistent with the topology inferred by ASTRAL-Pro. The divergence time was estimated by MCMCTREE with four fossils. The blue bars on the nodes are the ranges of 95% credibility interval (CI). The first row of the blocks on the top means the eras, while the second row of the blocks on the top means the periods. **B** Completeness of protein sets of each species assessed by applying the BUSCO (Insecta set) pipeline. **C** Network of the eight species based on the OGs. The size of the nodes represents the protein counts of the protein sets of eight species and the width of the edges represents the number of times two species occur in the same OGs. **D** Venn diagram of shared OGs in the transcriptome of *Icerya aegyptiaca* and three genomes

Protein sets from genomes or transcriptomes of six species from different neococcoid families and one outgroup aphid genome (Table 1) were used to assign the orthologous groups (OGs) together with the transcriptome of *I. aegyptiaca* by OrthoFinder [41], as the dataset for evolution analysis in the scale insects (Fig. 2A). Among the protein sets of all the eight species, complete genes evaluated by BUSCO [40] of 7 species account for more than 92%, except *E. pela* (87.6% complete genes), ensuring high integrity of the gene resources for further analysis (Fig. 2B). Totally 12,995 OGs were detected, including 320 species-specific OGs (supplemental Excel table). 5,446 OGs were present in all species and 2,514 OGs were single-copy among the eight species. According to the network of the shared OGs, protein sets from three genomes (*Acyrthosiphon pisum*, *M. hirsutus* and *E. pela*) contained fewer genes, while those protein sets from transcriptomes had more putative “genes” (Fig. 2C), which may be associated with the gene replications in transcriptomes caused by alternative splicing. Furthermore, protein sets of *I. aegyptiaca* and *Paratachardina pseudolobata* had the most proteins, which may be relevant to the size of the sequencing data used for assembly. The outgroup *A. pisum* shares the fewest OGs with other species. The shared OGs between *I. aegyptiaca* and other species from neococcoids are also relatively few, but comparatively large amounts of shared OGs among six neococcoid species are found, which conforms to putative genetic distances according to the taxonomy.

**Table 1:** Genomes and transcriptomes of scales and aphid for comparison and evolution analysis

ID	Family	Species	Data type	Database	Accession	Reference
APISU	Aphididae (outgroup)	*Acyrthosiphon pisum*	genome	InsectBase 2.0	IBG_00009	[42]
ACLER	Aclerdidae	*Aclerda sp.*	transcriptome	NCBI SRA	SRR1821892	[43]
EPELA	Coccidae	*Ericerus pela*	genome	https://datadryad.org/stash/share/ dIas3tyCEDAD1jJnCpZ6S7ZsqI 9WFMLN7QiasQBOlR4	-	[44]
DCONF	Dactylopiidae	*Dactylopius confusus*	transcriptome	NCBI SRA	SRR1821914	[43]
AUMBO	Diaspididae	*Acutaspis umbonifera*	transcriptome	NCBI SRA	SRR1821893	[43]
PPSEU	Kerriidae	*Paratachardina pseudolobata*	transcriptome	NCBI SRA	SRR5320111	[45]
MHIRS	Pseudococcidae	*Maconellicoccus hirsutus*	genome	InsectBase 2.0	IBG_00526	[46]
IAEGY	Monophlebidae	***Icerya aegyptiaca***	transcriptome	NCBI SRA	**SRR21713828-831**	**this study**

### Species phylogeny

Protein sequences of 2,514 single-copy OGs among 8 species were aligned, trimmed and concatenated into a protein supermatrix with the total length of 1,542,035 amino acids (aa), and then simplified using MARE (https://www.zfmk.de/en/research/research-centres-and-groups/mare) into a protein supermatrix made up by 848 OGs with 434,622 aa. Using IQ-TREE [47], 848 OGs were divided into 33 partitions and the best substitution models were estimated to get the phylogeny relationship among 8 species (Fig. 2A). The UFBoot values of the nodes are all 100, indicating high support values. Furthermore, the phylogenetic relationship is completely in accordance with the tree produced by the coalescent model of ASTRAL-Pro [48] with 905 highly supported OG trees (all nodes with UFBoot values >  = 80), which is also consistent with the position of each family in the previous study [49], indicating the reliability of the species tree.

According to the time tree estimated by MCMCTREE [50], the most recent common ancestor (MRCA) of aphids (Aphidomorpha) and scale insects (Coccomorpha) originated at 312.32 Ma (95% confidence interval (CI): 282.40–327.79 Ma) in Carboniferous (Fig. 2A). The divergence of *I. aegyptiaca* and neococcoids may occur at 247.14 Ma (95% CI: 221.87–266.95 Ma) in Triassic. Additionally, the neococcoids originated in the Early Cretaceous (137.39 Ma, 95% CI: 123.39–149.24 Ma).

### Comparison of genes in *I. aegyptiaca* and neococcoids

By summarizing the gene counts of each OG, we found that a large number of OGs were only present in our transcriptome of *I. aegyptiaca* (57,168, including 56,501 singletons) and absent in protein sets from three genomes of neococcoids and aphid (Fig. 2D), indicating large differences in genes from the data of *I. aegyptiaca* and neococcoids. We also explored the group-specific OGs (presence and absence) in *I. aegyptiaca* or neococcoids in our data. These presence and absence might be caused by actual emergence or loss, low expression and misassembly in the transcriptomes, thus we only provided them as a rough consultation (Table S1, detail in supplemental Excel table). Details of the GO enrichment analysis of these genes were provided in Figure S1-S4.

**Fig. 3:**
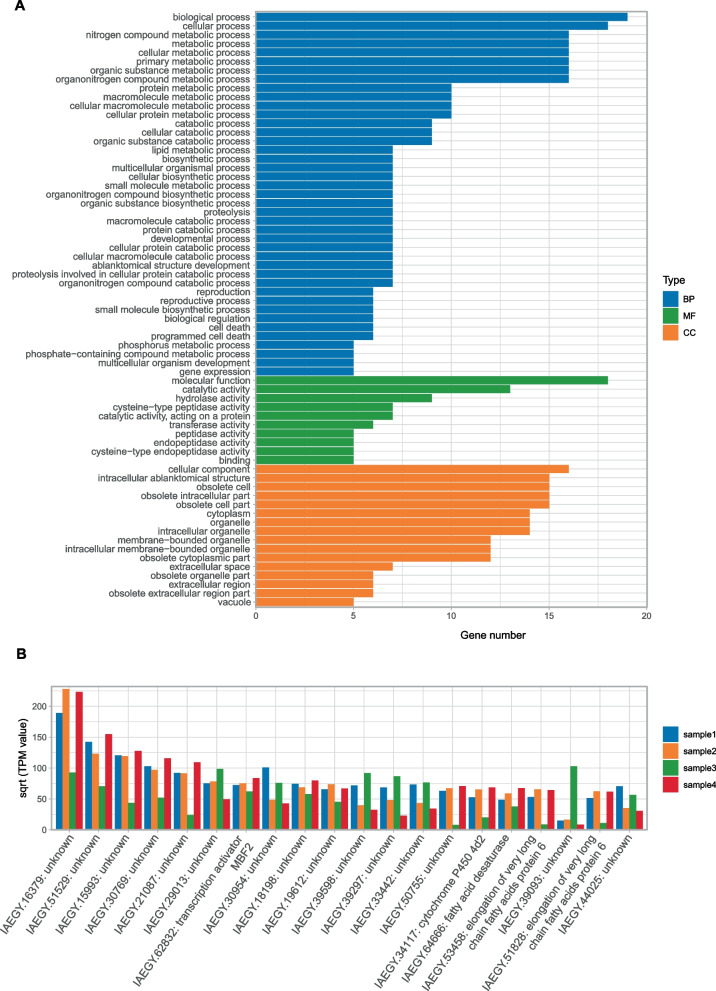
Highly expressed genes unique in *Icerya aegyptiaca*. **A** The Gene Ontology (GO) terms of 216 genes with average transcripts per kilobase million (TPM) values >  = 100 unique in *Icerya aegyptiaca*. Only the GO terms with gene counts >  = 5 were shown. BP: Biological Process category, MF: Molecular Function category, CC: Cellular Component category. **B** Top 20 unique genes in transcriptome of *I. aegyptiaca* with highest average TPM expression values

Further, we explored the highly expressed genes unique in *I. aegyptiaca* but not found in neococcoids, which are likely to indeed emergent in *I. aegyptiaca* with functional importance. Totally 216 genes (212 OGs) with average transcripts per kilobase million (TPM) values >  = 100 in four samples were found. These genes were mostly related to cellular metabolic, catabolic and biosynthetic process, and reproduction, development in Biological Process (BP) category, and catalytic, hydrolase and peptidase activity in Molecular Function (MF) category (Fig. 3A). Among the top 20 genes unique in *I. aegyptiaca* with highest average TPM values, 15 genes were with unknown function and 3 genes play roles in fatty acid desaturation or elongation (Fig. 3B).

### Positive selection and negativeselection relaxation at *I. aegyptiaca* or neococcoids

Branch-site model in CODEML [50] and BUSTED method in HyPhy [51] were both used to identify the OGs under positive selections. Totally 35 OGs at the node of *I. aegyptiaca*, 47 OGs at the node of neococcoid ancestor and 43 at the whole neococcoids clades were found under positive selection (supplemental Excel table).

The OGs selected positively at *I. aegyptiaca* were significantly enriched in metabolic process, neurogenesis, organ morphogenesis and transcription (Figure S5). Specifically, OG0004676 was annotated as Casein kinase I isoform epsilon (Table 2), which has roles in DNA replication and repair in the silkworm [52]. Ets DNA-binding protein pokkuri (OG0004313) was positively selected both in *I. aegyptiaca* and neococcoid ancestor and enriched under several terms in GO enrichment analysis. This gene is a regulator of photoreceptor, and proposed to be essential for the early development of compound eyes in *Drosophila* [53]. OG0006586 was also under positive selection. It was annotated as Tau-tubulin kinase homolog Asator, a tau-tubulin kinase interacts directly with the spindle matrix protein, and distributes to the spindle region during mitosis [54].

**Table 2:** Candidate orthologous groups (OGs) related to eye development and reproductive systems detected in selection pressure analysis in scale insects

OG	Branch	Selection	CODEML *P*-value	HyPhy *P*-value	Swiss-Prot annotation	Gene name	Putative function
OG0004676	*I. aegyptiaca*	Positive selection	< 0.001	0.033	Q5ZLL1	Casein kinase I isoform epsilon	DNA replication and repair [52]
OG0004313	*I. aegyptiaca*	Positive selection	< 0.001	0.003	Q01842	Ets DNA-binding protein pokkuri	early development of compound eyes [53]
OG0006586	*I. aegyptiaca*	Positive selection	< 0.001	0.046	Q8IMC6	Tau-tubulin kinase homolog Asator	activation of spindle in mitosis [54]
OG0004844	Neococcoid ancestor	Positive selection	0.007	0.021	A1Z6E0	Protein Gustavus	Arthropod reproduction [55]
OG0004313	Neococcoid ancestor	Positive selection	< 0.001	0.026	Q01842	Ets DNA-binding protein pokkuri	early development of compound eyes [53]
OG0006586	Neococcoid ancestor	Positive selection	< 0.001	0.033	Q8IMC6	Tau-tubulin kinase homolog Asator	activation of spindle in mitosis [54]
OG0005323	Neococcoid ancestor	Positive selection	< 0.001	0.046	Q99MY8	Histone-lysine N-methyltransferase ASH1L	H3K36 methylation [56]; development, gametogenesis and neurogenesis [57]
OG0005533	Neococcoid ancestor	Positive selection	< 0.001	< 0.001	Q9P2D1	Chromodomain-helicase-DNA-binding protein 7	Chromatin remodeling and H3K27 methylation [58]
OG0006511	Neococcoid clade	Positive selection	< 0.001	0.032	Q7KTX8	Mediator of RNA polymerase II transcription subunit 13	eye-antennal disc development [59]
OG0005570	Neococcoid clade vs *I. aegyptiaca*	Selection intensification	< 0.001	< 0.001	Q9QZR5	Homeodomain-interacting protein kinase 2	eye development [60]
OG0004591	Neococcoid clade vs *I. aegyptiaca*	Selection intensification	0.002	< 0.001	O12944	DNA repair and recombination protein RAD54-like	mitotic DNA repair and meiotic recombination [61]
OG0004840	Neococcoid clade vs *I. aegyptiaca*	Selection intensification	0.008	0.012	Q6DFM1	SWI/SNF-related matrix-associated actin-dependent regulator of chromatin subfamily B member 1	Chromatin remodeling [62]
OG0004516	Neococcoid clade vs *I. aegyptiaca*	Selection relaxation	< 0.001	< 0.001	Q99MQ1	Protein bicaudal C homolog 1 *Bic-C*	oogenesis and oocyte maturation [63]
OG0004594	Neococcoid clade vs *I. aegyptiaca*	Selection relaxation	< 0.001	0.015	P48608	Protein diaphanous	cytokinesis [64]

For positive selection, detection was performed by branch-site model in CODEML and BUSTED method in HyPhy. For selection intensification or relaxation, detection was performed by branch model in CODEML and RELAX method in HyPhy

The OGs positively selected at the ancestor of neococcoids were significantly enriched in embryo development, chromatin remodeling, and transcription (Figure S6). Among these OGs, OG0004844 was annotated as protein Gustavus (Table 2), which has a critical role in the reproductive success of a diverse number of arthropods [55]. OG0005323 and OG0005533 are related to chromatin and methylation, which are involved in H3K36 and H3K27 methylation respectively and possibly associated with development, gametogenesis and neurogenesis [56–58]. OG0004313 and OG0006586 was also under positive selection, similar with those in *I. aegyptiaca*, with the functions described above.

The OGs positively selected under the whole clade of neococcoids were mainly enriched in response to chemical, development, transport, and localization (Figure S7). We found mediator of RNA polymerase II transcription subunit 13 (OG0006511) was under positive selection in the whole clade of neococcoids (Table 2), which is required for identical processes in eye-antennal disc development [59].

In addition, 49 OGs were selected more intensively at whole neococcoid clade compared with *I. aegyptiaca*, and 71 OGs with negative selection relaxation, which were detected using both branch-model in CODEML [50] and RELAX method in Hyphy [51] (supplemental Excel table). GO enrichment showed that OGs mainly related to development, transport, chromatin, and response to compounds were selected with strengthened pressure in neococcoids (Figure S8). Homeodomain-interacting protein kinase 2 (OG0005570) was found under more intensive selection at whole neococcoid clade (Table 2), which interacts with Twin of eyeless (Toy) and is essential for proper eye development in fruit fly [60]. DNA repair and recombination protein RAD54-like (OG0004591), one of the major regulators of homologous recombination repair pathway that facilitates the steps of strand invasion in *Drosophila* [61] and SWI/SNF-related matrix-associated actin-dependent regulator of chromatin subfamily B member 1 (OG0004840), which contributes to regulating gene expression by adapting the structure of chromatin [62], were also under selection intensification in neococcoids. OGs related to epithelial cell differentiation, homeostasis, development and morphogenesis, transport, and respiratory system were under selection relaxation in neococcoids (Figure S9). One detected OG, annotated as protein bicaudal C homolog 1 *Bicaudal*-C (*Bic-C*) (OG0004516), confirmed to be required for oogenesis and oocyte maturation in *Nilaparvata lugens* [63] was found (Table 2). Besides, OG0004594 was annotated as protein diaphanous, which is required for cytokinesis [64].

### Common sex-specific differentially expressed genes within neococcoids

**Fig. 4:**
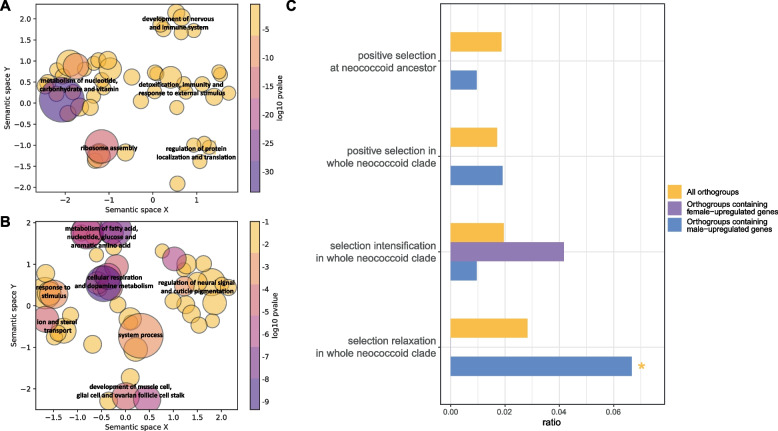
Common sex-specific differentially expressed genes (DEGs) in both *Maconellicoccus hirsutus* and *Ericerus pela*. The scatterplots of the enriched GO terms in Biological Process (BP) category with a similar function of (**A**) orthologous groups (OGs) containing female-upregulated genes; (**B**) OGs containing male-upregulated genes. The circles represent top 50 clusters of GO terms and the function descriptions are summaries according to the functions of these clusters. **C** The ratio of target OGs with evolution events in neococcoids in all OGs, OGs containing female-upregulated genes or containing male-upregulated genes. The orange asterisks mean that the ratio is significantly higher than the ratio in all OGs based on chi-square distribution, while the purple asterisks and blue asterisks mean that the ratio is significantly higher than the ratio in OGs containing female-upregulated or male-upregulated genes, respectively. * *P* < 0.05

There are 286 and 483 OGs containing upregulated differentially expressed genes (DEGs) in the female and male respectively, of both *M. hirsutus* and *E. pela* (supplemental Excel table). The common sex-specific DEGs significantly upregulated in the female neococcoids, were mainly associated with development of nervous and immune system, metabolism of nucleotide, carbohydrate and vitamin, detoxification, immunity and response to external stimulus, ribosome assembly, and regulation of protein localization and translation (Fig. 4A). In the male neococcoids, upregulated genes were related to metabolism of fatty acid, nucleotide, glucose and aromatic amino acid, response to stimulus, cellular respiration and dopamine metabolism, regulation of neural signal and cuticle pigmentation, ion and sterel transport, development of muscle cell, glial cell and ovatian follicle cell stalk, and system process (Fig. 4B). In the evolution of neococcoids, there were larger ratio of female-upregulated genes under intensified pressure selection than the male-upregulated genes and the whole OGs in the clade of neococcoids compared to *I. aegyptiaca*, while the ratio of male-upregulated genes under selection relaxation was higher than the whole OGs (*P* < 0.05) and female-upregulated genes (Fig. 4C). At neococcoid ancestor and neococcoid clade, the ratio of positively selected genes had no significant differences between the whole OGs, male and female-specific upregulated genes.

### Horizontally transferred genes in scale insects

**Fig. 5:**
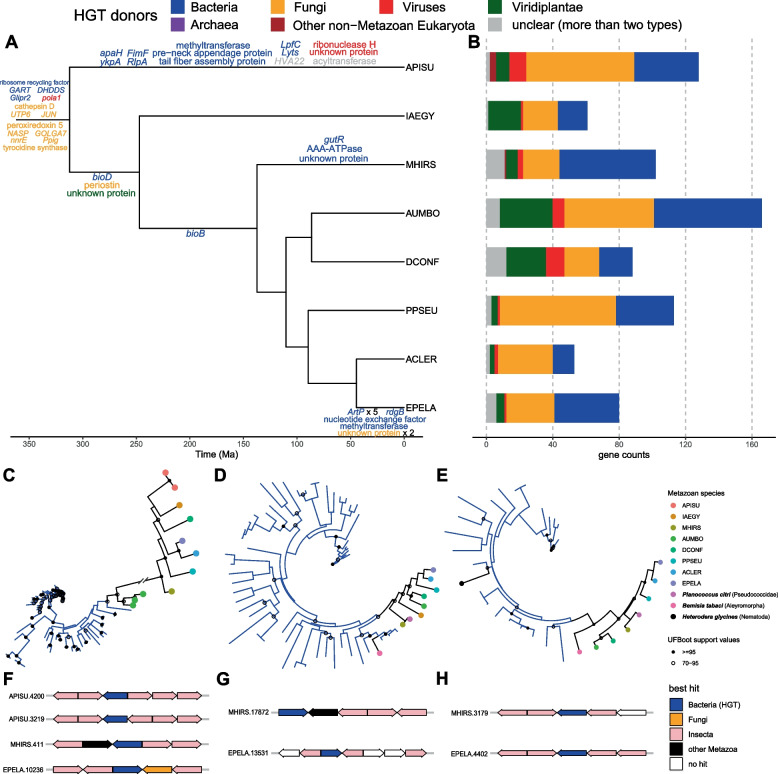
Putative horizontally transferred genes (HTGs) in seven scale insects and one outgroup aphid species. **A** Group-specific HTGs transferred to each node of the time tree. Different colors of the HTG names represent different putative horizontal gene transfer (HGT) donors. **B** Gene counts of HTGs from different putative donors in each species. Phylogeny of OG of HTGs and their homologous sequences in non-redundant (NR) database of (**C**) ribosome recycling factor; (**D**) *bioD*; (**E**) *bioB*. Black branches represent phylogeny of HTGs in Metazoan species, and branches with other colors mean phylogeny of homologous sequences in potential donors. Flank gene arrangements in three genomes of (**F**) ribosome recycling factor; (**G**) *bioD*; (**H**) *bioB*

We identified 791 candidate HTGs (from 92 OGs) in the genomes and transcriptomes of these 8 species (Fig. 5A), and the potential donors of these HTGs were mainly bacteria and fungi, among which bacteria account for 36.28% and fungi account for 39.82% (supplemental Excel table). A total of 61 HTGs were identified in *I. aegyptiaca*, of which the potential donors were mainly bacteria (29.51%), fungi (34.43%), viridiplantae (32.79%), viruses (1.64%) and unclear (more than two types) genes (1.64%) (Fig. 5B).

Totally 14 group-specific HTGs were found in all 8 species and thus identified to be transferred to the ancestor not later than MRCA of Aphidomorpha and Coccomorpha, among which, 4 genes were putative to be bacterial origin, including ribosome recycling factor, trifunctional purine biosynthetic protein adenosine-3 (*GART*), undecaprenyl diphosphate synthase complex subunit (*DHDDS*) and golgi-associated plant pathogenesis-related protein 1 (*Glipr2*). There were 9 genes whose potential donors were fungi, such as cathepsin D, tyrocidine synthase and NAD(P)H-hydrate epimerase (*nnrE*). In addition, one HTG from virus, the DNA polymerase alpha subunit A (*pola1*), was found (Fig. 5A). And there were three genes that seem to undergo horizontal transfer at the ancestor of Coccomorpha, including *bioD* whose potential donor is bacteria, periostin gene whose potential donor is fungi, and the unknown gene with Viridiplantae as the potential donor (Fig. 5A). At the ancestor of the neococcoids, the group-specific HTG was *bioB*, whose potential donor was bacteria, without detection in transcriptome of *I. aegyptiaca* (Fig. 5A).

The phylogenetic reconstruction of three widely existing HTGs in the scale insects, ribosome recycling factor, *bioD* and *bioB* showed that these three genes were indeed horizontally transferred from bacteria (Fig. 5C, 5D, 5E). These genes were all surrounded by the genes with putative insect origin, which excluded the possibility of contamination (Fig. 5F, 5G, 5H). Among these three HTGs, phylogenies of *bioD* and *bioB* of the scale insect species were similar with the species tree, with relatively high UFBoot support values (> = 70), especially at the node of scale insect HTG clade of *bioB*, which was higher than 95 (Fig. 5C, 5D, 5E). The respective monophyly of these three HTGs in the scale insects indicated that they were supposed to be of single origins, instead of potential contamination in those genes from the transcriptomes without flank genes to check. Additionally, *bioD* also exists in *Planococcus citri* (Pseudococcidae) and *Bemisia tabaci* (Aleyromorpha) according to the homologous sequences from non-redundant (NR) database, while *bioB* exists in *P. citri*, *B. tabaci* and *Heterodera glycines* (Nematoda). However, the HTGs in the Metazoan species might be dispersed in different clades and were likely to be obtained through independent horizontal gene transfer (HGT) events. In the tree of *bioD*, the HTG from the whitefly *B. tabaci* was not contained in the clade of scale insect HTGs (Fig. 5D). And the *bioB* gene of *H. glycines* was not in the same clade as *bioB* genes in the hemipteran species, which may not arise from the same horizontal gene transfer events (Fig. 5E).

## Discussion

### Potential genetic link to the unique structures in the scale insects

In this study, we provided first transcriptome of *I. aegyptiaca*, and by comparison with the genomes and transcriptomes of seven species, many genes related to development were detected from the evolution analyses, revealing some secrets of the unique structures of scale insects.

First, we found that genes related to the development of compound eyes, respiratory system, or sensory organs go through positive selection and negative selection intensification in *I. aegyptiaca*, along with some eye development-related genes selected in neococcids, including Ets DNA-binding protein pokkuri [53]. Actually, the anatomies of scale insects like eyes, spiracles and antenna display a high diversity, which have long been used as standards for classification [65]. Most representatively, compound eyes in males and abdominal spiracles are absent in neococcoids, but present in most other scale insects, including Iceryini [6, 12, 13]. Although the character of compound eyes only appears in the male, the related genes may be expressed and found in the female transcriptome with a relatively low expression. It is probably that these evolving genes caused the diversity of these structures, at least those except compound eyes.

In addition, the surface of *I. aegyptiaca* is relatively complex, with much structures developed, including extensions developed from the ventral surface of the abdomen, circatrix and derm pores producing the secreted substance [66]. Furthermore, the adult female *I. aegyptiaca* is covered with lumps of white waxy secretions, together with 20 waxy processes surrounding the body margin [66]. In our study, we could find the potential connections that the OGs related to epithelial cell differentiation and development were positively selected or negatively selected with strengthened pressure at *I. aegyptiaca*, while some genes related to fatty acid biosynthesis and elongation were unique and highly expressed in the *I. aegyptiaca* transcriptomes and not found in other scale insects. These fatty acid-related genes are likely to be emergent genes in the lineage of *I. aegyptiaca*, which is a powerful genetic explanation for the abundance wax in *I. aegyptiaca* and partly accounting for its widely invasion due to the protective function [32].

### Genetic clues of reproductive system formation: hermaphrodite and PGE

Scale insects exhibits several types of reproductive system, including hermaphroditism and PGE. It has been confirmed the presence of hermaphroditism in Iceryini, which is the only case known in insects [17]. Here, we found that the gene related to DNA repair (Casein kinase I isoform epsilon [52]) in *I. aegyptiaca* was positively selected, which possibly leads to functional evolution in repair process and influence DNA replication during the cells division. This may lead to its abnormal way of cell division for producing both egg and sperm. At the same time, the genes related to cytokinesis (protein diaphanous [64]) and oogenesis (*Bic-C* [63]) was under negative selection intensification, which tends to make the gene conservative and keep its importance during cell division and germ cell formation respectively. These genes may be the candidate genes that are involved in reproduction of *I. aegyptiaca* and help form its unique hermaphrodite system, and deserve to be further explored.

PGE is another magical reproductive determine system in insect, which has a single origination at the ancestor of neococcoids in the scale insects [6]. Recent study has shown that several factors such as mitosis, inverted meiosis, and DNA methylation play significant roles in the evolution of PGE The combination of holokinetic chromosomes and inverted meiosis in mites had been confirmed to be relative to PGE-like systems [16]. The failure of normal pairing between homologous chromosomes, which reduces recombination between maternal and paternal chromosomes, may also be involved in the evolution of PGE [19]. Studies of the mealybug *Planococcus citri* and *Phenacoccus solenopsis* (Hemiptera: Pseudococcidae) have found that they both lack differentiated sex chromosomes but reveal sex-specific genomic imprinting [67, 68]. In *P. solenopsis*, H3K9me3 and H3K27me3 were distributed widely in males, and may be the epigenetic basis of PGE in mealybugs [67]. Our study similarly found that genes under positive selection or negative selection intensification in neococcoids were enriched in chromatin-related processes, such as chromatin remodeling, suggesting that chromatin changes may play vital roles in the PGE system. Notably, the genes involved in H3K36 and H3K27 methylation were detected to be positively selected at the neococcoid ancestor, among which H3K27 methylation was reported as critical role in PGE system of *P. solenopsis* [67] as mentioned above. Several genes related to meiosis and mitosis were alsoselected in neococcoid ancestor, such as Tau-tubulin kinase homolog Asator that are required for the normal activation of spindle involving in mitosis [54, 69], and DNA repair and recombination protein RAD54-like that takes part in mitotic DNA repair and meiotic recombination [61], indicating that the performance of meiosis and mitosis in neococcoids underwent a potential change, probably from primitive XX-XO to PGE system in their ancestor. Mitotic division has been confirmed important in another insect with PGE system, *Liposcelis booklice* (Insecta: Psocodea), that have several mitotic divisions at the end of spermatogenesis [70], which supports our results. More experimental evidences in the PGE system are further required for these chromatin and division-related genes.

### Sex-specific genes in neococcoids and impact of PGE system

Under the PGE system, however, similar with other scales insects, the male adult neococcoids possess wings after maturity, while female adults remain instar-like and immobile. The common sex-specific DEGs in neococcoids in our study exhibited these great sexual dimorphisms, with genes related to muscle cell development, respiration and juvenile hormone binding upregulated in the male adults, which are associated with their wing development [9, 71, 72], and genes related to detoxification, immunity, response to external stimulus and metabolism of carbonhydrate and vitamin upregulated in the female adults, which seems to be associated with management and digestion of the food. However, since the data used in our study was from the adult stage, these DEGs may be related to not only sex development, but also other mechanisms.

In addition, these common sex-specific DEGs show potential connections with the evolution events of neococcoids. Those male-specific upregulated genes tend to undergo negative selection relaxation, while the female-specific upregulated genes seem to be easier to go through negative selection intensification (though not significant) in the neococcoid clade. Females and males always face different selection pressures, even in haplodiploids like neococcoids [73, 74]. In aphid with XX-XO system, unexpressed genes have the most relaxed selection pressure, followed by male-biased genes, and then female-biased genes and finally unbiased genes, which is possibly related to the frequency of the gene exposure and less existence of male aphids [74]. Similarly, it seems that neococcoids with PGE systems have rarer males than females, thus leading to selection relaxation in male-biased genes. Additionally, elimination of paternal genome impedes the inheritance of the selection imprints from the males, which may be an even more important factor.

### Horizontally transferred genes and symbiotic relationship evolution in the scale insects

We detected the HTGs in the scale insects in this study and found large amounts of HTGs, mainly from bacteria or fungi. This is similar with what previous study found in whole insect lineage [75], but our results indicate less HTGs from bacteria (36.28%) and more HTGs from fungi (39.82%) in scale insects than all insects (~ 80% and ~ 11%, respectively). Many reported HTGs are also found in our study, such as *RlpA* in *A. pisum* [76–78], *bioB* and *bioD* in *M. hirsutus* [27], which can complement missing symbiont genes for cell wall and biotin synthesis.

Among these HTGs, interestingly, we found that, the ribosome recycling factor, which encodes one of key translational control proteins in bacteria, existed in all eight species of scale insects and aphids, implying that this gene was transferred from bacteria into the ancestor not later than the root of our time tree, that is, the MRCA of Aphidomorpha and Coccomorpha. However, no homologous sequences from other Hemipteran or even Metazoan were found in NR database, and only the bacterial sequences were similar, thus validating the HGT event of this gene. The major function of ribosome recycling factor is to release mRNA from the post-termination complex of the ribosome [79]. But this gene is lost in all genomes of *Tremblaya*, the symbiotic bacteria of the mealybugs (Pseudococcidae) [27]. It seems reasonable that this gene can also complement the missing function of translational control in the mealybugs, and even all the scale insects. Nevertheless, this gene exists in the aphid *A. pisum*, but the obligate symbiotic bacteria *Buchnera* of the aphids still remains ribosome recycling factor [80], indicating a different function from complementing the missing function of obligate symbiotic bacteria.

In addition, *bioD* and *bioB*, the two genes reported as biotin synthesis HTGs in mealybugs and whiteflies [23, 27, 81, 82], were found transferred from bacteria into ancestor of scale insects and neococcoids respectively in our study. At the same time, Flavobacteria probably settled down in the scale insects and then left from some neococcoids [25, 26], which may active the demand of *bioD* and *bioB*. But another biotin synthesis HTGs *bioA* that is always reported together [23, 27, 81, 82] is not detected as group-specific HTGs at any internal nodes of our scale insect phylogeny. These two genes are responsible for the last two steps of biotin synthesis and participate in the missing part of biotin synthesis pathway of symbiotic bacteria of mealybugs and whiteflies, thereby supplying the vitamin to the host insects [23, 82]. Extending to the whole scale insects, it is possible to be a common symbiotic relationship based on our findings. Although both scale insects and whiteflies belong to Sternorrhyncha, our tree of *bioD* revealed that the HTG in *B. tabaci* and the HTGs in the scale insects were obtained from close bacterial donors through independent HGT events. The absence of this gene in the sister group, the aphid *A. pisum*, supports this inference. Similarly, *bioB* of the scale insects and the nematode were likely to come from different donors through different HGT events, but the genes of the scale insects and *B. tabaci* were clustered into a monophyletic clade. However, *bioB* is only found in neococcoid species and not detected in the transcriptome of the sister group *I. aegyptiaca* and the genome of the closer group *A. pisum* than the whitefly. Actually, HTGs may evolve following the insect genome adaptation, which lead to some advolution [75], we hence tend to consider that *bioB* of the scale insects and the whitefly are not acquired in the same HGT event.

Apart from these potential biotin synthesis HTGs related to complement of nutritional pathway, however, no cell wall synthesis genes were identified as HTGs at any internal nodes in our scale insect phylogeny. Those cell wall synthesis HTGs, such as *RlpA*, tend to disperse in different species. This is similar with the discovery in the mealybug lineage, in which the cell wall synthesis HTGs are always acquired later, and the ancient HTGs are related to nutrition synthesis [27].

## Conclusions

In this study, we sequenced and assembled the first transcriptome of *I. aegyptiaca*, which can provide a representative genetic resource for this species, and even non-neococcoid scale insects. Together with several species from different neococcoid families, we further constructed the evolutionary framework of scale insects. Through our analysis, the evolution events of the genes may be connected to the development of unique structures and the peculiar reproductive systems in *I. aegyptiaca* and neococcoids. And the HTGs in the scale insects indicate the potential complement functions to the symbiotic bacteria. However, it should be noticed that transcriptomes in several species were used in the comparison, which would cause inaccuracy due to unexpressed genes, redundancy of alternative splicing and misassembly, especially at the aspect of gene counts. The transcriptomes also prevent the insight into the evolution of gene expansions, contractions, and rearrangements in the scale insects. In addition, large amounts of genes in *I. aegyptiaca* have no functional information, which may be the key genes for its interesting hermaphrodite system. Therefore, it deserves to increase the genomes of the scale insects from different families and conduct experimental studies on their gene functions in the future.

## Materials and methods

### RNA extraction and transcriptome sequencing

The female adults of *I. aegyptiaca* were wild collected from the *Litsea monopetala* (Roxb.) tree in Sun Yat-sen University, Guangzhou, China. Four individuals were selected and each female adult was used as one sample for transcriptome sequencing, then frozen in liquid nitrogen before RNA extraction. The total RNA of each individual was extracted using TRIzol reagent (CWBIO, Beijing, China), separately. RNA quality and quantity were determined using a Nanodrop 1000 spectrophotometer (Thermo Fisher Scientific, Wilmington, USA). Only RNA samples with a 260/280 ratio from 1.8 to 2.0, a 260/230 ratio from 2.0 to 2.5 and an RNA integrity number (RIN) greater than 8.0 were used for sequencing. Sequencing was performed on the Illumina HiSeq 2500 platform with a 6-Gb sequencing depth for each sample, generating 2 × 125 bp (Base pairs) reads.

### Transcriptome assembly, protein set construction and functional annotation

Adaptors and low-quality sequences were first removed from the raw reads using Trimmomatic v0.36 [83] with the default settings. The transcriptomes of *I. aegyptiaca* individuals were then together de novo assembled by Trinity v2.8.4 [33]. In consideration of the frequent contamination in the transcriptomes, we downloaded all the nucleotide sequences under Coccoidea (Taxonomy ID: 33,381) from the National Center for Biotechnology Information (NCBI) nucleotide sequence (NT) database. Subsequently, the transcriptome assembly of *I. aegyptiaca* were searched against both the Coccoidea database and the NCBI NT database by BLAST v2.8.1 + [84]. Those transcriptomic sequences with percent identity < 90 to the sequences from the Coccoidea database and percent identity >  = 98 to the sequences from the NCBI NT database were identified as foreign sequences and removed from the assembly. Further, the clean assembly were submitted to EvidentialGene v2018.06.18 [34] to construct non-redundant coding gene sets. With the coding sequences (CDSs) as reference, the abundance of coding genes in each sample was quantified by RSEM v1.3.1 [85] with Bowtie2 v2.2.5 [86] as the aligner. The proteins from the gene sets were annotated by a modified “annotate” pipeline of FunAnnotate v1.8.1 (https://github.com/nextgenusfs/funannotate) with the same procedures as Tang et al. (2022) [87]. Using this method, the GO [39] annotations were obtained by combining the results of InterProScan v5.32 (InterPro v71.0 database) [35, 36] and Eggnog-mapper v2.0.0 (EggNog v5.0 database) [37, 38].

### Data collection and orthologous group assignment

Genomes or transcriptomes of six scale species from different neococcoid families and one outgroup aphid species were collected from InsectBase 2.0 database [88], NCBI Sequence Read Archive (SRA) database or published supplementary (Table 1). The transcriptomic protein sets of *Aclerda* sp., *Dactylopius confusus*, *Acutaspis umbonifera* and *P. pseudolobata* were constructed using the same method as that of *I. aegyptiaca*. The protein sets of *A. pisum* and *M. hirsutus* were obtained directly from the gene predictions provided by InsectBase, while the protein set of *E. pela* was predicted using the modified FunAnnotate pipeline with the same procedures as Tang et al. (2022) [87] with RNA-Seq data in different life stages and sex (SRA accession: SRR9617903, SRR9617905, SRR9617904, SRR9617907, SRR9617913 and SRR9617918) [89]. All the protein sets were annotated using the modified “annotate” pipeline of FunAnnotate with the same method as *I. aegyptiaca*, and then evaluated by BUSCO v3.0.2 pipeline [40] with the Insecta set of OrthoDB v9 database [90].

The OGs between the eight protein sets were assigned by OrthoFinder v2.3.3 [41]. The information of the OG assignment was summarized by KinFin v1.0.3 [91] and the network of eight species based on the OGs was plotted by Cytoscape v3.9.0 [92]. The GO and other annotations of the OGs were obtained from the consensus on the annotations of each gene member in the OGs, and only the annotations of >  = 75% members were kept. Additionally, the OGs were aligned using L-INS-i mode of MAFFT v7.427 [93] and then respectively searched against UniProtKB/Swiss-Prot v2020_05 database [94] by HMMER v3.2.1 [95] with the E-value cutoff of 1e-5. The best result of each OG was considered as the Swiss-Prot annotation.

### Phylogenetic analysis

Protein sequences of 2,514 single-copy OGs among the eight species identified by OrthoFinder were aligned using L-INS-i mode of MAFFT v7.427 [93] and the alignments were trimmed by trimAl v1.4 [96] with the “automated1” option, separately, which aimed to remove the poorly aligned regions. Then the concatenated supermatrix of 2,514 alignments was reduced by MARE v0.1.2-rc (https://www.zfmk.de/en/research/research-centres-and-groups/mare) with the default settings, and then imported into the “MFP + MERGE” mode of IQ-TREE v2.1.2 [47] to search for the best partition scheme and the best models and reconstruct the phylogeny of the eight species with 1,000 ultrafast bootstrap (UFBoot) replicates. Additionally, all the OGs were aligned by MAFFT and trimmed by trimAl as same as the single-copy OGs, and then used to reconstruct the OG trees by IQ-TREE with 1,000 UFBoot replicates. The OG trees with all internal nodes which had UFBoot support values >  = 80 were selected and input to ASTRAL-Pro v1.1.6 [48] to generate a species tree under the coalescent model. Finally, the species tree was rooted with the aphid species *A. pisum* using the Python ETE 3 Toolkit [97].

Divergence time estimation was then conducted by using MCMCTREE in PAML v4.8a package [50]. The uncorrelated rate model (clock = 2) was used, and four fossils were chosen to calibrate the clock (Table 3). Referring to estimated time of MRCA of Aphidomorpha and Coccoidea in TimeTree database [98], 251 million years ago (Ma) was set as the root age to calculate the prior on the overall substitution rate with the CODEML program in the PAML package under the JTT + F + GAMMA model with four rate categories. Subsequently, we constrained the maximum bound for the root (MRCA of Aphidomorpha and Coccomorpha) to 323.2 Ma, which is the beginning of the Pennsylvanian, the epoch without any record of hemipteran fossil in the EDNA fossil insect database [99]. The ML estimates of the branch lengths were also calculated by CODEML using 33 partitions from the protein supermatrix divided by IQ-TREE. The gamma-Dirichlet prior for overall rate for genes (rgene gamma) was set at G (1, 5.81), and the gamma-Dirichlet prior for sigma^2 (sigma2 gamma) was set at G (1, 4.5). Then, the MCMCTREE program in PAML estimated the divergence times by using a Monte Carlo algorithm. Burn-in was set as 1,000,000, meaning the first 1,000,000 iterations were discarded. Next, the samples were recorded every 1,000 iterations until 10,000 samples were obtained. Another run was started from different seeds following the same steps. Finally, to check the convergence of the result, we used Tracer v1.7.2 [100] to ensure that all parameters were similar between two runs and that their effective sample sizes (ESSs) were all larger than 200.

**Table 3:** Information of fossils used for calibration points in the species phylogenetic analysis

Calibrated node	Note	Fossil taxa	Minimum age	Reference
MRCA of Aphidomorpha and Coccomorpha (root)	*Vosegus triassicus*	Triassoaphidoidea—Vosegidae	238	[101]
Crown Coccomorpha	*Eomatsucoccus andrewi*	Coccoidea—Matsucoccidae	127	[102]
MRCA of neococcoid families	*Williamsicoccus megalops*	Coccoidea—Pseudococcidae	124	[103]
MRCA of Aclerdidae and Coccidae	*Rosahendersonia prisca*	Coccoidea—Coccidae	93.5	[103]

*MRCA* Most recent common ancestor

### Enrichment of group-specific orthologous group

To observe the whole condition of the gene function, the group-specifically present and absent OGs in transcriptomes of *I. aegyptiaca* and the data of neococcoids were used to perform the GO enrichment by hypergeometric distribution testing through clusterProfiler package [104], respectively. For *I. aegyptiaca*-specific OG presence, we only considered the OGs containing genes with all transcripts per kilobase million (TPM) values >  = 1 in four samples, which are likely to be functionally important and unlikely to be contamination, while the genes with average TPM values >  = 100 were considered as highly expressed genes and used for display in the main results. All the OGs including the singletons were used as background. *P*-values were further adjusted for multiple testing using Benjamini–Hochberg Procedure without the cutoffs in order to observe all the enriched terms.

### Selection pressure detection of single-copy orthologous groups

Using trimAl v1.4 [96], the protein alignments of 2,514 single-copy OGs were directly used as the blueprint to generate the CDS alignments. At the same time, the CDS alignments were trimmed for selection pressure detection. To avoid potential false positive in the detection, both CODEML of PAML v4.8a [50] and HyPhy v2.5.0 [51] were used for the detection. Only the OGs with *P*-values less than 0.05 obtained by both software were considered as significant results.

We used the branch-site model (parameters: null hypothesis: model = 2, NSsites = 2, fix_omega = 1, omega = 1; alternative hypothesis: model = 2, NSsites = 2, fix_omega = 0, omega = 1) in CODEML and BUSTED method in HyPhy to identify the positively selected genes (genes with positively selected sites) (PSGs) at the *I. aegyptiaca* node, neococcoid ancestor node, and all nodes within the neococcoid clade together, respectively. The target node or nodes were labeled as the foreground branch and then likelihood ratio tests (LRTs) were performed to detect positive selection on the foreground branch or branches.

Furthermore, the relaxation and intensification of selection pressure of *I. aegyptiaca* compared with branches within neococcoid clade were detected by the branch model (model = 2, NSsites = 0) in CODEML and RELAX method in HyPhy. For alternative hypothesis in CODEML, the branches of *I. aegyptiaca* and neococcoid clade were labeled with #1 and #2, respectively, and branches of other branches were considered as background branches. For null hypothesis in CODEML, both the branches of *I. aegyptiaca* and neococcoid clade were labeled with #1. Then the LRTs were conducted based on the likelihood values of models of alternative hypothesis and null hypothesis. When dN/dS value of the branch of *I. aegyptiaca* was larger than that of the branches within neococcoid clade in alternative hypothesis calculation, it was considered as selection relaxation in *I. aegyptiaca* or selection intensification in neococcoid clade, while when dN/dS of the branch of *I. aegyptiaca* was smaller, it was thought to be as selection intensification in the branch of *I. aegyptiaca* or selection relaxation in neococcoid clade. For RELAX method, the branch of *I. aegyptiaca* was set as the test branch set, and the branches within neococcoid clade was set as reference branch set.

The GO enrichments on the detected OGs under positive selection or selection intensification/relaxation was conducted by clusterProfiler package [104] without the cutoffs in order to observe all the enriched terms, using all the single-copy OGs as background.

### Neococcoid sex-specific differentially expressed gene identification and analysis

To identify the common sex-specific DEGs within neococcoids, we collected the transcriptomes of female and male adults of *M. hirsutus* (female: SRR13590443, SRR13590444; male: SRR13590441, SRR13590442) [46] and *E. pela* (female: SRR1027687, SRR1027688, SRR1027689; male: SRR1027692) [71]. The downloaded reads were then mapped to the genomes of *M. hirsutus* or *E. pela* using HISAT2 v2.2.1 [105]. Abundance estimation was performed using StringTie v2.1.4 [106] based on genome annotation. Differential expression analysis between treatments was performed using edgeR package [107] according to the standard workflow, with a log2(fold change) (log2FC) value > 1 or < -1 and an adjusted *P*-value (Q value) < 0.05 used as the criteria for defining DEGs. Since the male adult group of *E. pela* had no replicates, only the log2FC value was considered and the results were interpreted carefully together with the DEGs in *M. hirsutus*. The OGs containing female-biased and male-biased DEGs in both *M. hirsutus* and *E. pela* were considered the common female-biased and male-biased DEGs within neococcoids, respectively.

The GO enrichments of the common female-specific and male-specific DEGs within neococcoids were performed by clusterProfiler package [104] with no cutoffs to observe all the enriched terms. The raw *P*-values were passed to GO-Figure! python package [108] to produce scatterplots of the enriched GO terms with similar function. To explore trends in evolutionary events at neococcoids on female-specific and male-specific DEGs, the ratio of the detected OGs compared with all OGs, OGs containing common female-biased or male-biased DEGs were calculated and compared using chi-square test in R.

### Horizontally transferred gene detection

All the proteins from the eight species were searched against the NCBI NR database by DIAMOND v2.0.9.147 [109] with default E-value and maximum target sequences of 500. The search results were submitted to the Alienness web server (http://alienness.sophia.inra.fr/cgi/tool.cgi) [110], with Metazoa (Taxonomy ID: 33,208) as taxonomic group of interest (ingroup) and Hemiptera (Taxonomy ID: 7524) as exclusion group to avoid homologous sequences in the close species. The likely HGTs identified by Alienness web server with alien index (AI) > 15 and percent identity to non-Metazoan sequences < 70 (to avoid misidentification due to the contamination) were considered as candidate HTGs. Subsequently, all the candidate HTGs with their matched hits from the NCBI NR database were aligned using L-INS-i mode of MAFFT v7.427 [93] and then used to build the gene trees by FastTree v2.1.11 [111]. According to these gene trees, we preliminarily identified the candidate HTGs, which were surrounded by their non-Metazoan donors.

In order to avoid the impact of potential contamination and inactive genes in the transcriptomes, the abundance of coding genes in each species was quantified using the same method as mentioned above for *I. aegyptiaca*, and only the candidate HTGs with TPM values of all samples >  = 1 were remained. In addition, we checked the origin of the genomic and transcriptomic sequences where the coding genes came, especially the flank regions of the target genes, based on the BLAST hits generated in foreign sequence identification when assembling the transcriptomes and DIAMOND search of the proteins mentioned above for the three genomes. First, those genomic sequences containing >  = 60% genes with best hits from non-Metazoan sequences and those transcriptomic sequences containing >  = 60% region with best hits from non-Metazoan sequences were considered as likely contamination, and the genes on them were excluded from the HGT detection. Other candidate genes were remained as the candidate HTGs. Then, the genes from those sequences containing >  = 60% genes or regions with best hits from Insecta sequences were identified as the putative HTGs. Only the OGs containing one or more putative HTGs were used in the downstream analyses, while other OGs only containing the candidate HTGs without exact Insecta origin were listed as consultation in the supplemental Excel table.

Finally, IQ-TREE v2.1.2 [47] was used to reconstruct the phylogeny using the alignments of all the corresponding OG members of putative HTGs and their best 30 matched hits from NR database with 1000 UFBoot replicates, and the HTGs were manually checked through visualization by MEGA v10.2.2 [112] and R package ggtree [113]. The taxonomy of the sequences within the two clades surrounding the target Hemiptera clade were considered as the potential HGT donors. The group-specifical OGs were tentatively considered as the HTGs transferred into these groups (potential HGT receptors).

## Supplementary Information

**Additional file 1:** **Table S1.** Group-specific orthologous groups (OGs) of *I. aegyptiaca* or neococcoids possibly related to the unique structures and reproductive systems in the scale insects. DEG: differentially expressed genes. M: *Maconellicoccus hirsutus*, E: *Ericerus pela*. **Figure S1.** The Gene Ontology (GO) enrichment of group-specifically present orthologous groups (OGs) in transcriptome of *I. aegyptiaca* with genes that have all transcripts per kilobase million (TPM) values >=1 in four samples. **Figure S2.** The Gene Ontology (GO) enrichment of group-specifically absent orthologous groups (OGs) in transcriptome of *I. aegyptiaca*. **Figure S3.** The Gene Ontology (GO) enrichment of group-specifically present orthologous groups (OGs) in neococcoids. **Figure S4.** The Gene Ontology (GO) enrichment of group-specifically absent orthologous groups (OGs) in neococcoids. **Figure S5.** The Gene Ontology (GO) enrichment of positively selected genes (PSGs) at *I. aegyptiaca*. **Figure S6.** The Gene Ontology (GO) enrichment of positively selected genes (PSGs) at neococcoid ancestor. **Figure S7.** The Gene Ontology (GO) enrichment of positively selected genes (PSGs) at whole neococcoid clade. **Figure S8.** The Gene Ontology (GO) enrichment of orthologous groups (OGs) with selection intensification at whole neococcoid clade compared with *I. aegyptiaca*. **Figure S9.** The Gene Ontology (GO) enrichment of orthologous groups (OGs) with selection relaxation at whole neococcoid clade compared with *I. aegyptiaca*.

## Data Availability

The RNA-Seq data of *Icerya aegyptiaca* were deposited in the NCBI BioProject: PRJNA883806 http://www.ncbi.nlm.nih.gov/bioproject/883806. Supplemental Excel table and supplemental sequence file were deposited in https://github.com/huangyh45/Icerya-aegyptiaca.
