# Supplementary material for: The transcriptome of Icerya aegyptiaca (Hemiptera: Monophlebidae) and comparison with neococcoids reveal genetic clues of evolution in the scale insects
Source: BMC Genomics. 2023 May 3;24:231. doi: 10.1186/s12864-023-09327-z (PMC10158165; doi:10.1186/s12864-023-09327-z)
Supplement: Supplementary file 1 — Additional file 1: Table S1. Group-specific orthologous groups (OGs) of I. aegyptiaca or neococcoids possibly related to the unique structures and reproductive systems in the scale insects. DEG: differentially expressed genes. M: Maconellicoccus hirsutus, E: Ericerus pela. Figure S1. The Gene Ontology (GO) enrichment of group-specifically present orthologous groups (OGs) in transcriptome of I. aegyptiaca with genes that have all transcripts per kilobase million (TPM) values >=1 in four samples. Figure S2. The Gene Ontology (GO) enrichment of group-specifically absent orthologous groups (OGs) in transcriptome of I. aegyptiaca. Figure S3. The Gene Ontology (GO) enrichment of group-specifically present orthologous groups (OGs) in neococcoids. Figure S4. The Gene Ontology (GO) enrichment of group-specifically absent orthologous groups (OGs) in neococcoids. Figure S5. The Gene Ontology (GO) enrichment of positively selected genes (PSGs) at I. aegyptiaca. Figure S6. The Gene Ontology (GO) enrichment of positively selected genes (PSGs) at neococcoid ancestor. Figure S7. The Gene Ontology (GO) enrichment of positively selected genes (PSGs) at whole neococcoid clade. Figure S8. The Gene Ontology (GO) enrichment of orthologous groups (OGs) with selection intensification at whole neococcoid clade compared with I. aegyptiaca. Figure S9. The Gene Ontology (GO) enrichment of orthologous groups (OGs) with selection relaxation at whole neococcoid clade compared with I. aegyptiaca. [file 12864_2023_9327_MOESM1_ESM.docx]

**Supplementary materials**

Table S1 Group-specific orthologous groups (OGs) of *I. aegyptiaca* or neococcoids possibly related to the unique structures and reproductive systems in the scale insects. DEG: differentially expressed genes. M: *Maconellicoccus hirsutus*, E: *Ericerus pela*

| OG | Group | Presence /absence | Sex-specific DEGs | Swiss-Prot annotation | Function description |
| --- | --- | --- | --- | --- | --- |
| OG0093756 | *I. aegyptiaca* | presence | - | Q9H7Z6 | Histone acetyltransferase KAT8, also called Mof (males absent on the first). An essential component of the X chromosome dosage compensation system in *Drosophila* (Thomas et al., 2008) |
| OG0007941 | *I. aegyptiaca* | absence | - | B1WAR9 | Serine/threonine-protein kinase greatwall. Required in *Drosophila* for proper chromosome condensation (Yu et al., 2004) |
| OG0007310 | *I. aegyptiaca* | absence | - | P0CY46 | Epidermal growth factor receptor. Involved in the determination of adult ovary development in honeybee (Formesyn et al., 2014) |
| OG0002409 | *I. aegyptiaca* | absence | - | O46339 | Homeobox protein homothorax. Prevent inappropriate eye development, carry homeodomain required for proper localization of chordotonal organs within the peripheral nervous system and antennal identity (Inbal et al., 2001) |
| OG0008636 | *I. aegyptiaca* | absence | - | Q9VRP9 | E3 ubiquitin-protein ligase Bre1. An E3 ubiquitin ligase required for the monoubiquitination of histone H2B and participated in H3K4 methylation (Bray et al., 2005) |
| OG0008669 | neococcoids | presence | E♀ | O08901 | Mitotic checkpoint serine/threonine-protein kinase BUB1. Required for the spindle checkpoint (Manic et al., 2017) |
| OG0007676 | neococcoids | presence | E♀ | P24862 | G2/mitotic-specific cyclin-B. May involve in oocyte maturation in honeybee (He et al., 2019); may be essential for oogenesis and the ovary development of *Plutella xylostella* (Peng et al., 2017) |
| OG0008943 | neococcoids | presence | E♂ | Q9VRP9 | E3 ubiquitin-protein ligase Bre1. Same as OG0008636 |
| OG0001461 | neococcoids | presence | M♂, E♀, E♂ | Q9EQQ0 | Histone-lysine N-methyltransferase. Related to heterochromatin, and may causes histone H3-lysine (H3K9) methylation in *Drosophila* (Peng and Karpen, 2007) |
| OG0005753 | neococcoids | presence | M♂, E♂ | Q9VBV3 | Protein takeout. Encode multiple factors with sex-specific function; Takeout/juvenile hormone binding proteins (TO/JHBP) involve in several important processes in insects (Dauwalder et al., 2002; Hagai et al., 2007) |
| OG0008170 | neococcoids | presence | M♂, E♂ | Q9VBV3 | Protein takeout. Same as OG0005753 |
| OG0011793 | neococcoids | absence | - | Q9D099 | alkaline ceramidase 3. Important for the development of male testis (Zhang et al., 2021) |

**Reference:**

Bray S, Musisi H, Bienz M. Bre1 is required for Notch signaling and histone modification. Dev Cell. 2005; 8(2): 279-286.

Dauwalder B, Tsujimoto S, Moss J, Mattox W. The *Drosophila* takeout gene is regulated by the somatic sex-determination pathway and affects male courtship behavior. Genes Dev. 2002; 16(22): 2879-2892.

Formesyn EM, Cardoen D, Ernst UR, Danneels EL, Van Vaerenbergh M, De Koker D, Verleyen P, Wenseleers T, Schoofs L, de Graaf DC. Reproduction of honeybee workers is regulated by epidermal growth factor receptor signaling. Gen Comp Endocrinol. 2014; 197: 1-4.

Hagai T, Cohen M, Bloch G. Genes encoding putative Takeout/juvenile hormone binding proteins in the honeybee (*Apis mellifera*) and modulation by age and juvenile hormone of the takeout-like gene GB19811. Insect Biochem Mol Biol. 2007; 37(7): 689-701.

He XJ, Jiang WJ, Zhou M, Barron AB, Zeng ZJ. A comparison of honeybee (*Apis mellifera*) queen, worker and drone larvae by RNA‐Seq. Insect Science, 2019; 26(3): 499-509.

Inbal A, Halachmi N, Dibner C, Frank D, Salzberg A. Genetic evidence for the transcriptional-activating function of Homothorax during adult fly development. Development. 2001; 128(18): 3405-3413.

Manic G, Corradi F, Sistigu A, Siteni S, Vitale I. Molecular regulation of the spindle assembly checkpoint by kinases and phosphatases. Int Rev Cell Mol Biol. 2017; 328: 105-161.

Peng JC, Karpen GH. H3K9 methylation and RNA interference regulate nucleolar organization and repeated DNA stability. Nat Cell Biol. 2007; 9(1):25-35.

Peng L, Wang L, Yang YF, Zou MM, He WY, Wang Y, Wang Q, Vasseur L, You MS. Transcriptome profiling of the *Plutella xylostella* (Lepidoptera: Plutellidae) ovary reveals genes involved in oogenesis. Gene. 2017; 637: 90-99.

Thomas T, Dixon MP, Kueh AJ, Voss AK. Mof (MYST1 or KAT8) is essential for progression of embryonic development past the blastocyst stage and required for normal chromatin architecture. Mol Cell Biol. 2008; 28(16): 5093-5105.

Zhang MJ, Shi XX, Wang N, Zhang C, Zhang C, Quais MK, Ali SA, Zhou W, Mao C, Zhu ZR. Transcriptional changes revealed genes and pathways involved in the deficient testis caused by the inhibition of alkaline ceramidase (Dacer) in *Drosophila melanogaster*. Arch Insect Biochem Physiol. 2021; 106(3): e21765.


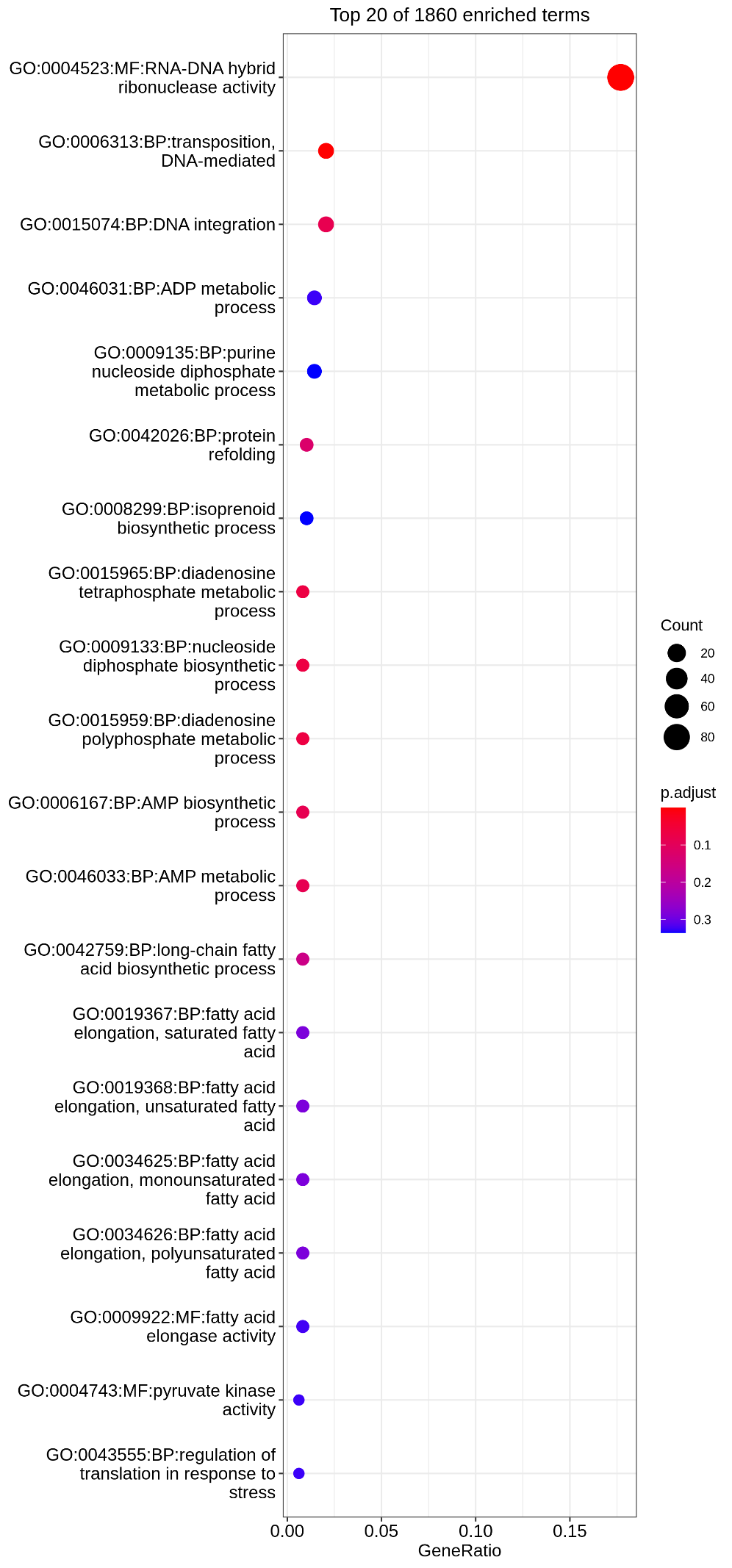


Figure S1 The Gene Ontology (GO) enrichment of group-specifically present orthologous groups (OGs) in transcriptome of *I. aegyptiaca* with genes that have all transcripts per kilobase million (TPM) values >=1 in four samples.


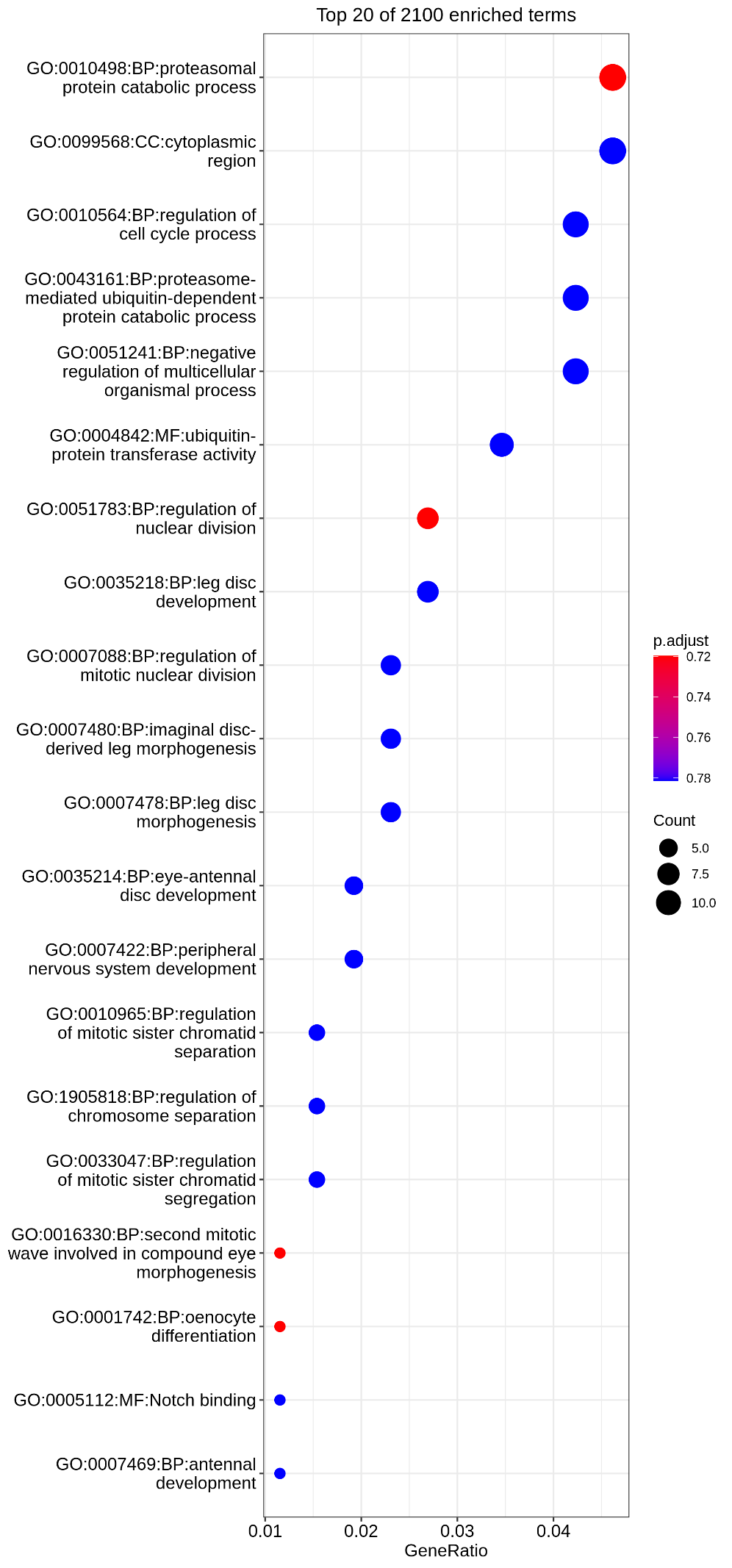


Figure S2 The Gene Ontology (GO) enrichment of group-specifically absent orthologous groups (OGs) in transcriptome of *I. aegyptiaca*.


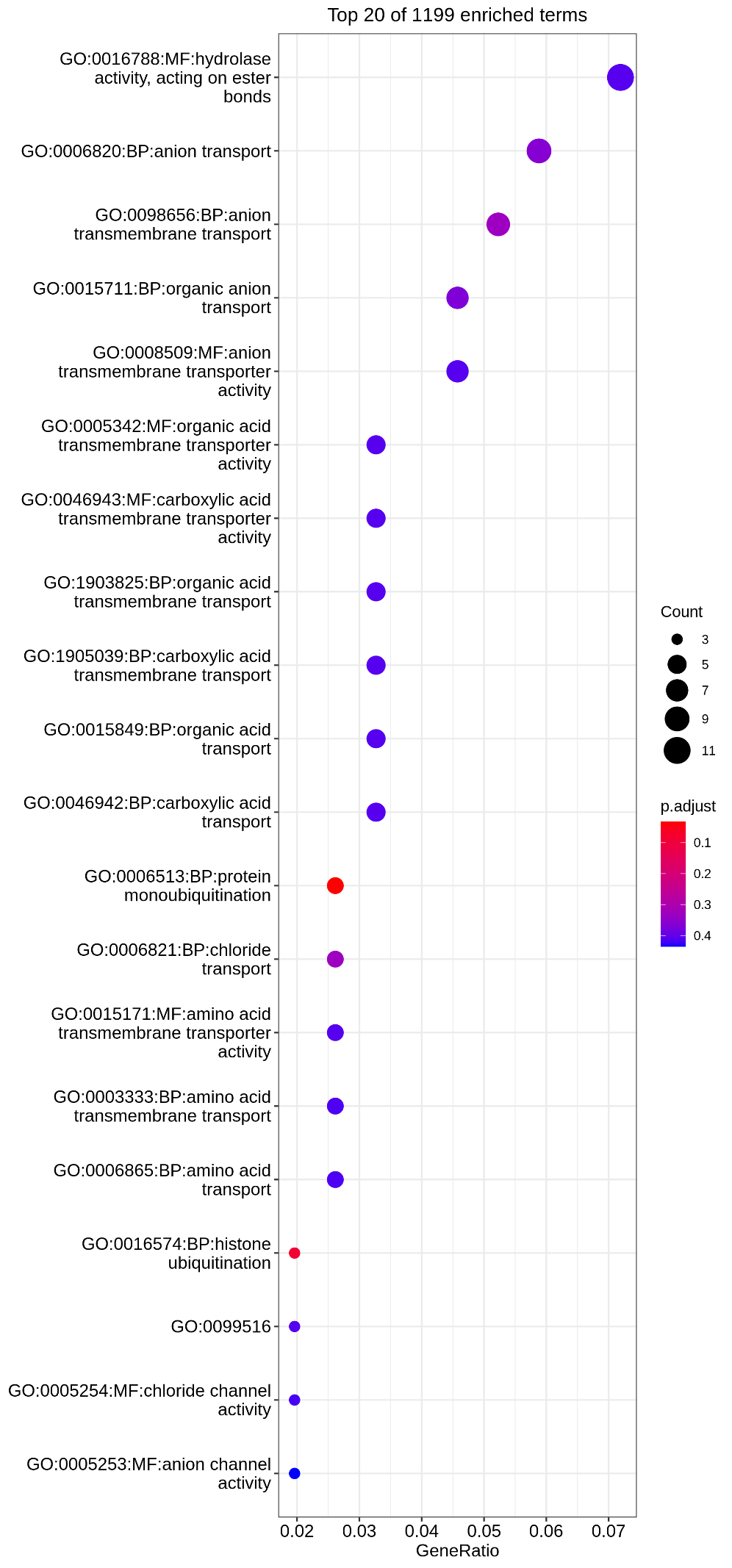


Figure S3 The Gene Ontology (GO) enrichment of group-specifically present orthologous groups (OGs) in neococcoids.


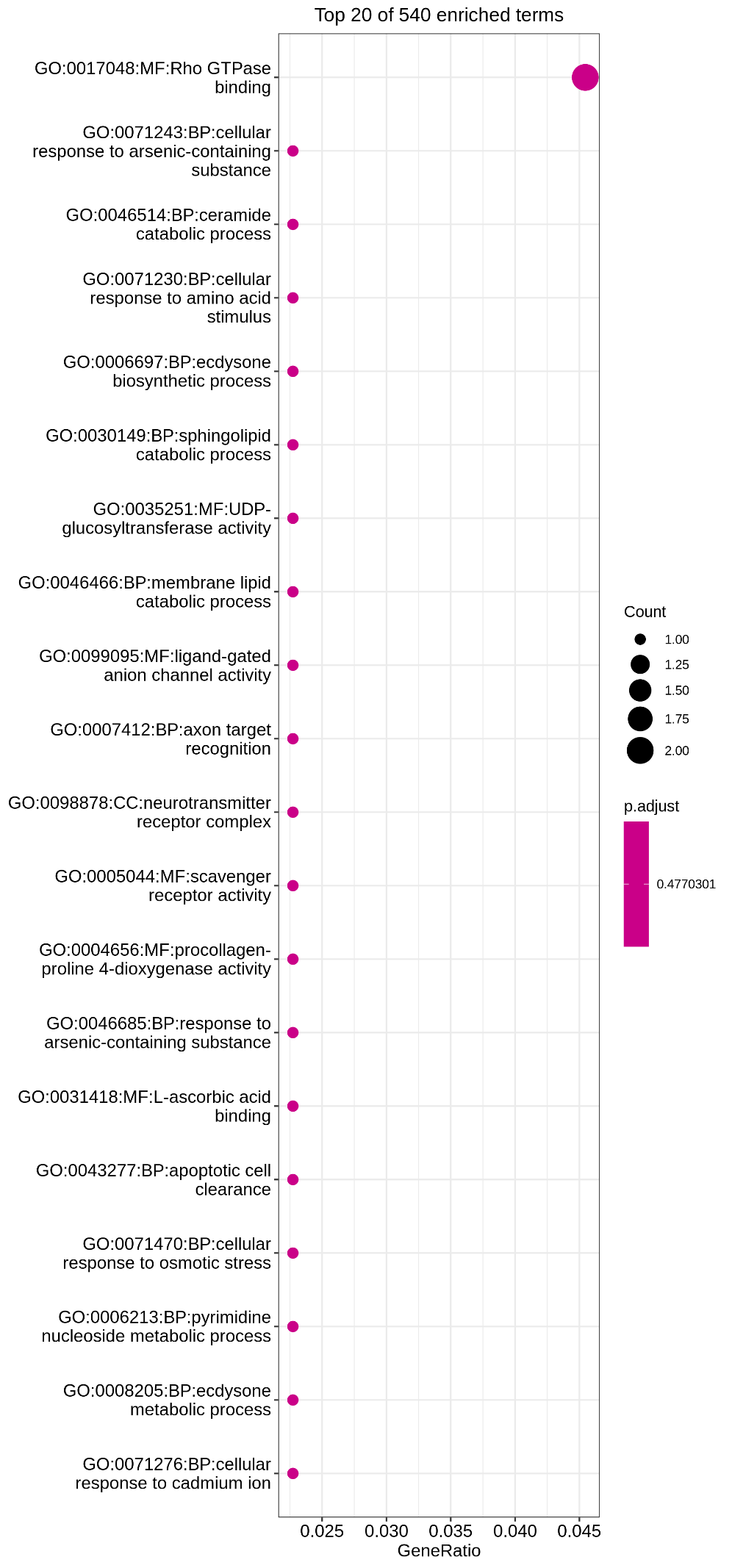


Figure S4 The Gene Ontology (GO) enrichment of group-specifically absent orthologous groups (OGs) in neococcoids.


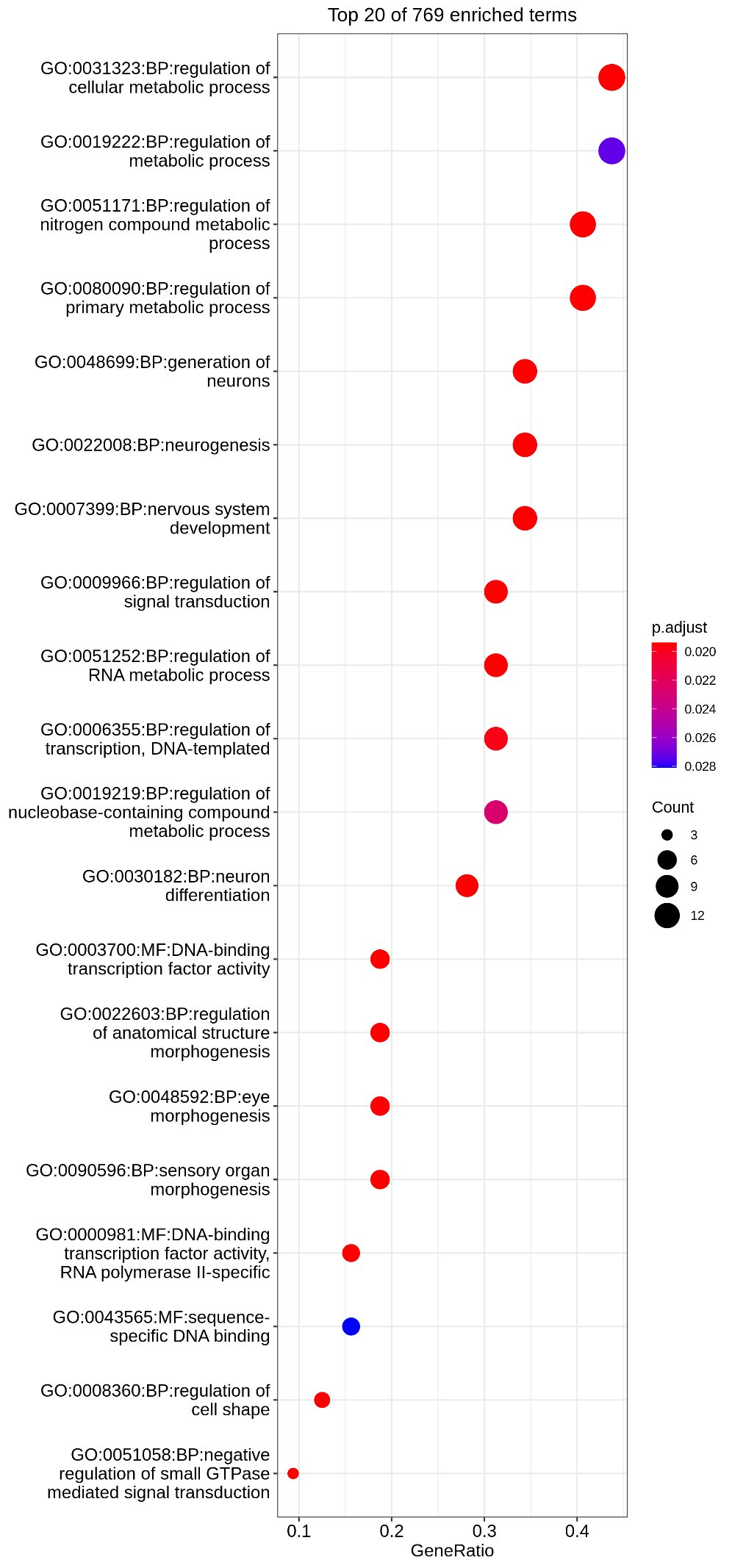


Figure S5 The Gene Ontology (GO) enrichment of positively selected genes (PSGs) at *I. aegyptiaca*.


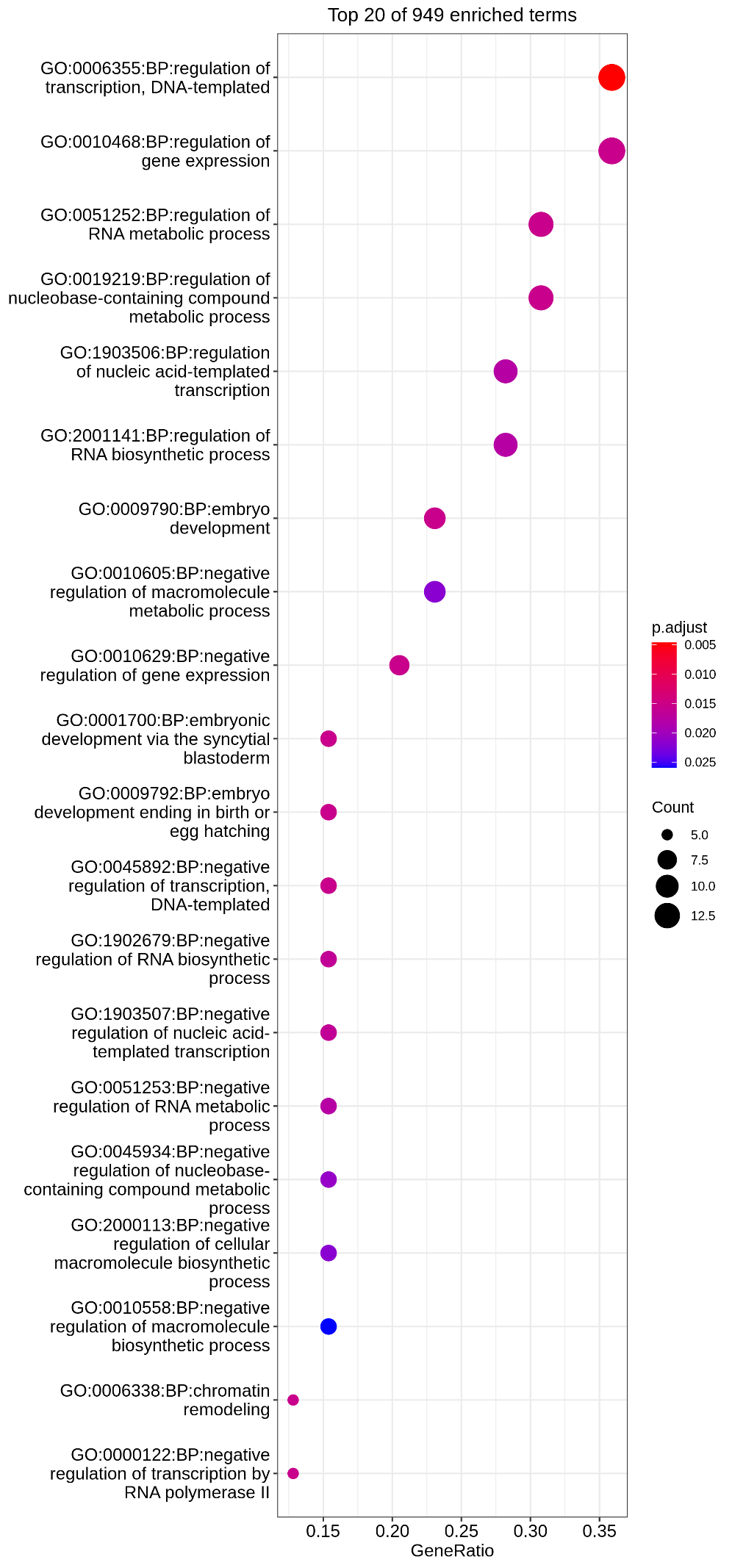


Figure S6 The Gene Ontology (GO) enrichment of positively selected genes (PSGs) at neococcoid ancestor.


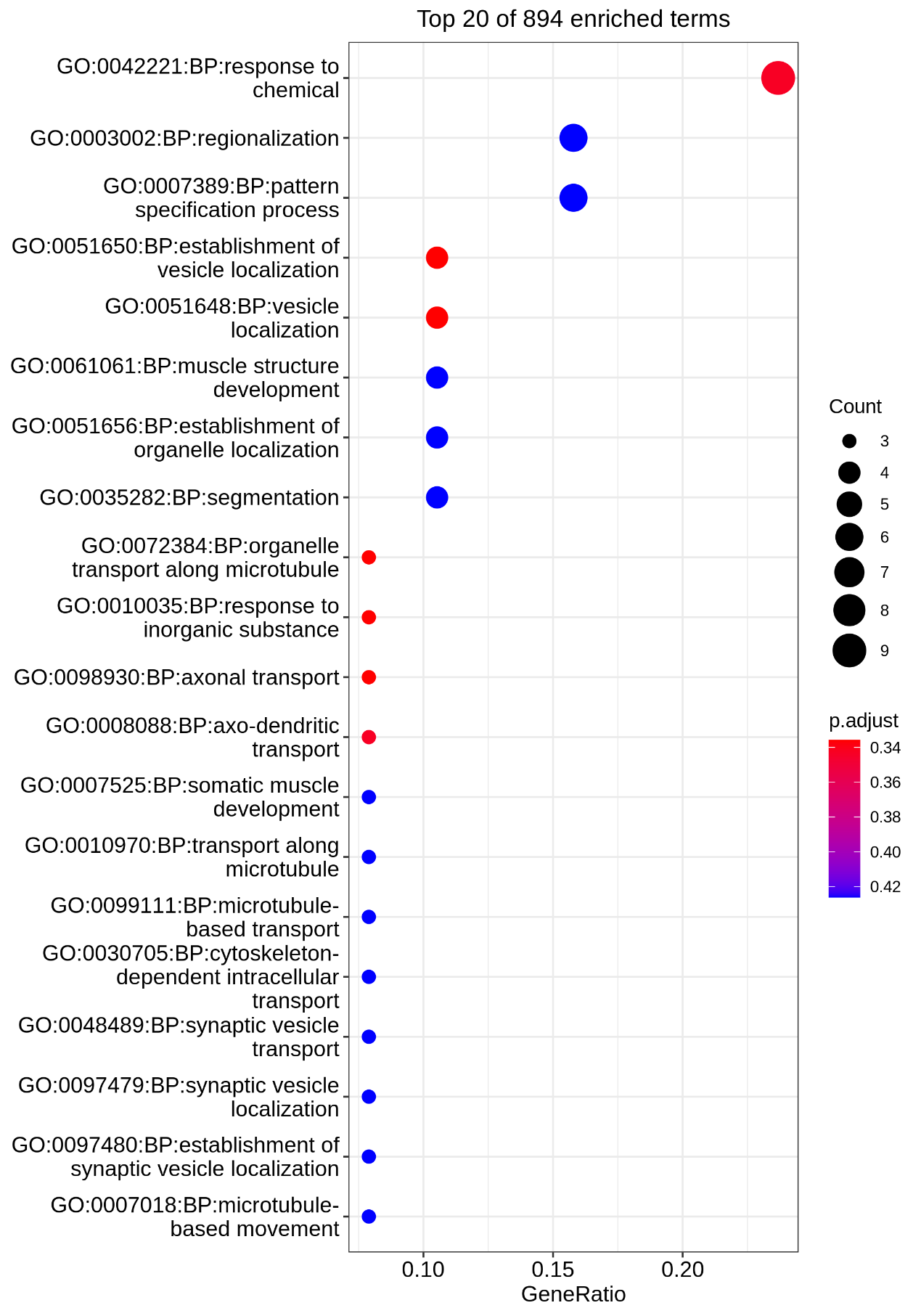


Figure S7 The Gene Ontology (GO) enrichment of positively selected genes (PSGs) at whole neococcoid clade.


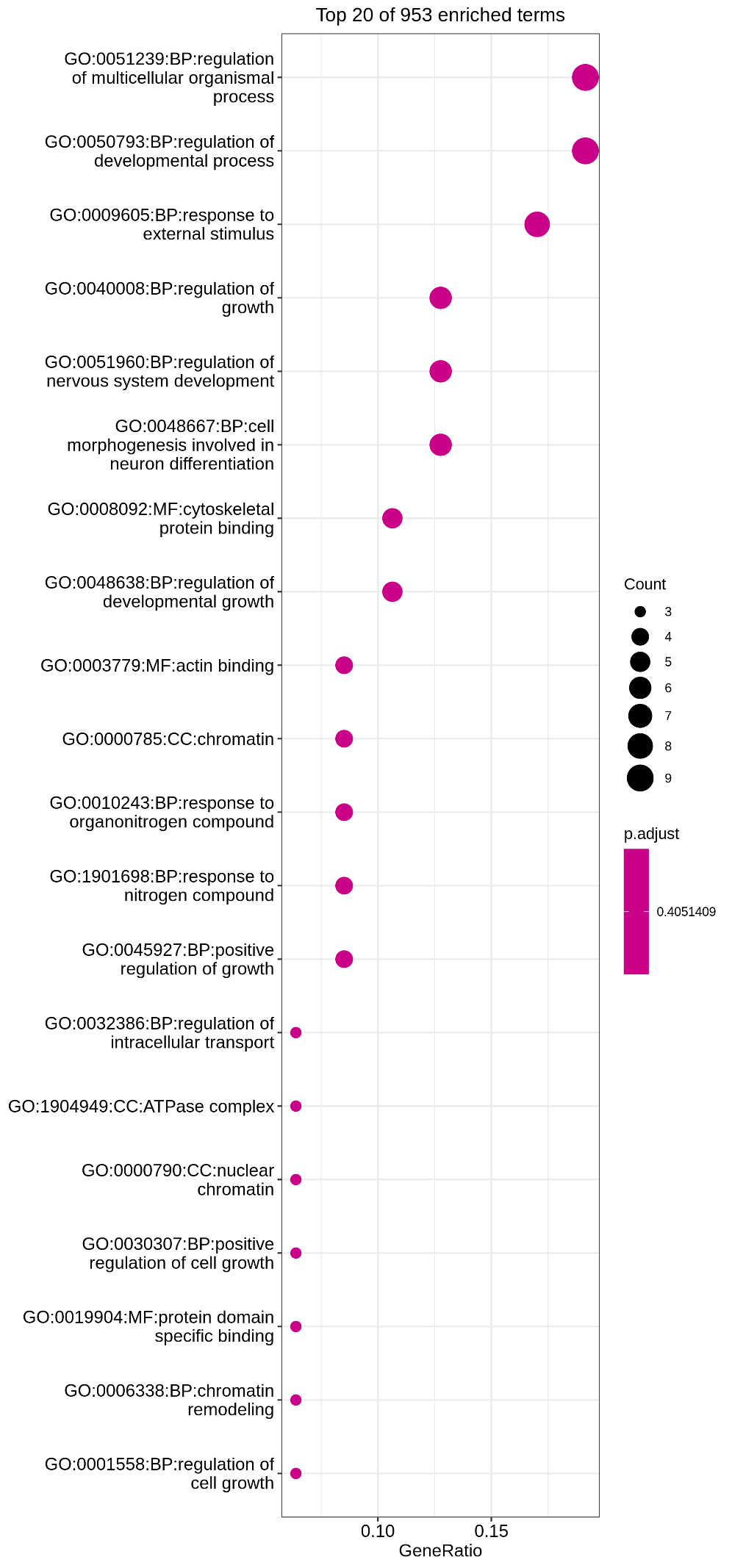


Figure S8 The Gene Ontology (GO) enrichment of orthologous groups (OGs) with selection intensification at whole neococcoid clade compared with *I. aegyptiaca*.


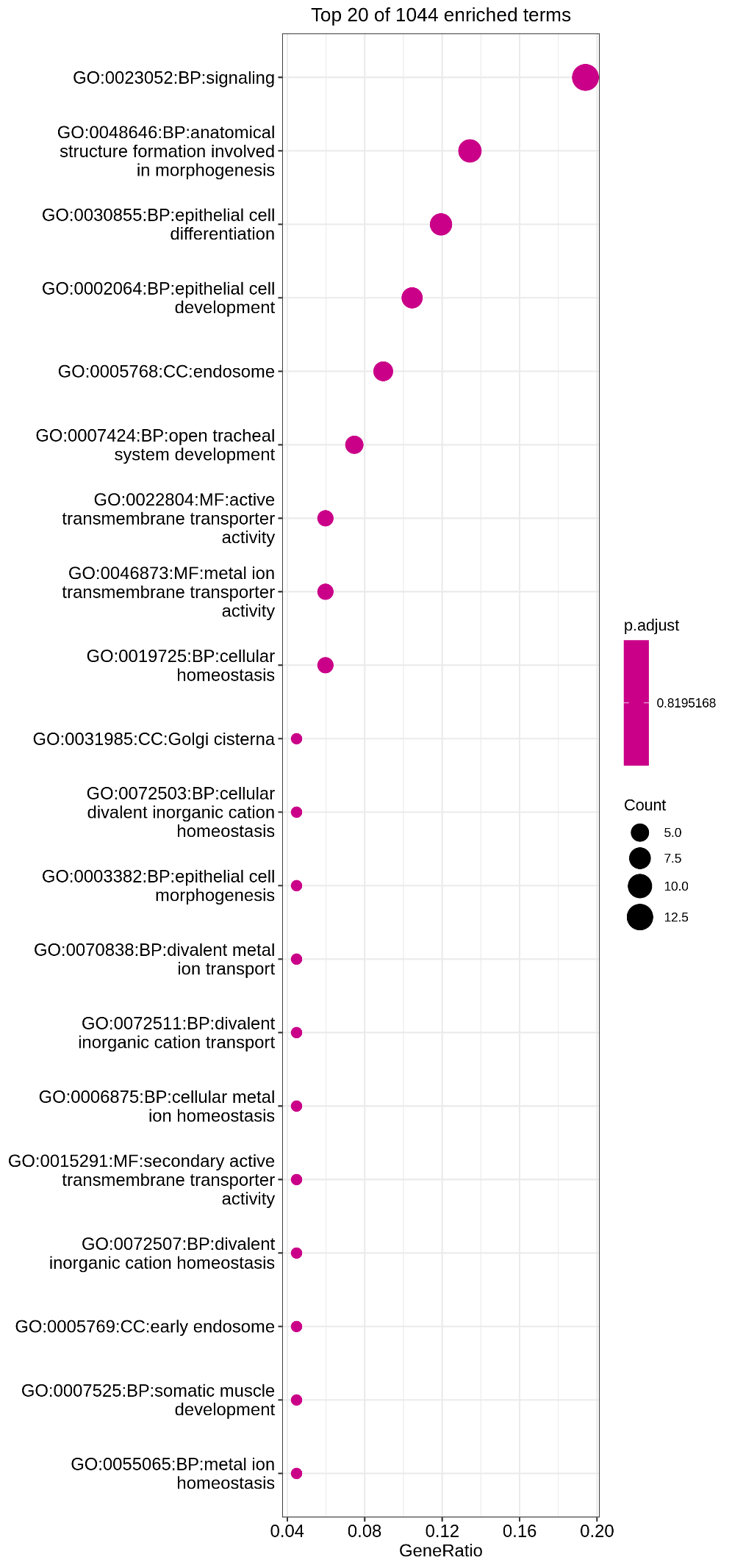


Figure S9 The Gene Ontology (GO) enrichment of orthologous groups (OGs) with selection relaxation at whole neococcoid clade compared with *I. aegyptiaca*.
